# Single-Cell Analyses Reveal Megakaryocyte-Biased Hematopoiesis in Myelofibrosis and Identify Mutant Clone-Specific Targets

**DOI:** 10.1016/j.molcel.2020.04.008

**Published:** 2020-05-07

**Authors:** Bethan Psaila, Guanlin Wang, Alba Rodriguez-Meira, Rong Li, Elisabeth F. Heuston, Lauren Murphy, Daniel Yee, Ian S. Hitchcock, Nikolaos Sousos, Jennifer O’Sullivan, Stacie Anderson, Yotis A. Senis, Olga K. Weinberg, Monica L. Calicchio, NIH Intramural Sequencing Center, Deena Iskander, Daniel Royston, Dragana Milojkovic, Irene Roberts, David M. Bodine, Supat Thongjuea, Adam J. Mead

**Affiliations:** 1 Haematopoietic Stem Cell Biology Laboratory, Medical Research Council (MRC) Weatherall Institute of Molecular Medicine (WIMM), University of Oxford, Oxford OX3 9DS, UK; 2 MRC Molecular Haematology Unit, MRC WIMM, University of Oxford, Oxford OX3 9DS, UK; 3 NIHR Biomedical Research Centre, University of Oxford, Oxford OX4 2PG, UK; 4 Hematopoiesis Section, National Human Genome Research Institute, National Institutes of Health, Bethesda, MD 20892-4442, USA; 5 MRC WIMM Centre for Computational Biology, MRC WIMM, University of Oxford, Oxford OX3 9DS, UK; 6 York Biomedical Research Institute and Department of Biology, University of York, Heslington, York YO10 5DD, UK; 7 NHGRI Flow Cytometry Core, National Human Genome Research Institute, National Institutes of Health, Bethesda, MD 20892-4442, USA; 8 Institut National de la Santé et de la Recherche Médicale Unité Mixte de Recherche-S 1255, Etablissement Français du Sang Grand Est, Strasbourg 67065, France; 9 Department of Pathology, Boston Children’s Hospital, Boston, MA 02115, USA; 10 National Institutes of Health, Bethesda MD 20892-442, USA; 11 Centre for Haematology, Hammersmith Hospital, Imperial College of Medicine, London W12 OHS, UK; 12 Nuffield Division of Clinical Laboratory Sciences, Radcliffe Department of Medicine, University of Oxford, Oxford OX3 9DS, UK; 13 Department of Paediatrics, University of Oxford, Oxford OX3 9DU, UK

**Keywords:** megakaryopoiesis, myeloproliferative neoplasm, platelets, TARGET-seq, immunotherapy, single-cell multi-omics, G6B, fibrosis, bone marrow

## Abstract

Myelofibrosis is a severe myeloproliferative neoplasm characterized by increased numbers of abnormal bone marrow megakaryocytes that induce fibrosis, destroying the hematopoietic microenvironment. To determine the cellular and molecular basis for aberrant megakaryopoiesis in myelofibrosis, we performed single-cell transcriptome profiling of 135,929 CD34^+^ lineage^−^ hematopoietic stem and progenitor cells (HSPCs), single-cell proteomics, genomics, and functional assays. We identified a bias toward megakaryocyte differentiation apparent from early multipotent stem cells in myelofibrosis and associated aberrant molecular signatures. A sub-fraction of myelofibrosis megakaryocyte progenitors (MkPs) are transcriptionally similar to healthy-donor MkPs, but the majority are disease specific, with distinct populations expressing fibrosis- and proliferation-associated genes. Mutant-clone HSPCs have increased expression of megakaryocyte-associated genes compared to wild-type HSPCs, and we provide early validation of G6B as a potential immunotherapy target. Our study paves the way for selective targeting of the myelofibrosis clone and illustrates the power of single-cell multi-omics to discover tumor-specific therapeutic targets and mediators of tissue fibrosis.

## Highlights

• Single-cell-omics demonstrate megakaryocyte-biased hematopoiesis in myelofibrosis (MF)
• Megakaryocyte progenitors (MkPs) show high expression of a fibrosis signature in MF
• MkPs are heterogeneous in MF with aberrant metabolic and inflammatory signatures
• Targeting aberrant surface G6B expression may selectively ablate the MF clone

## Graphical Abstract

**Figure:**
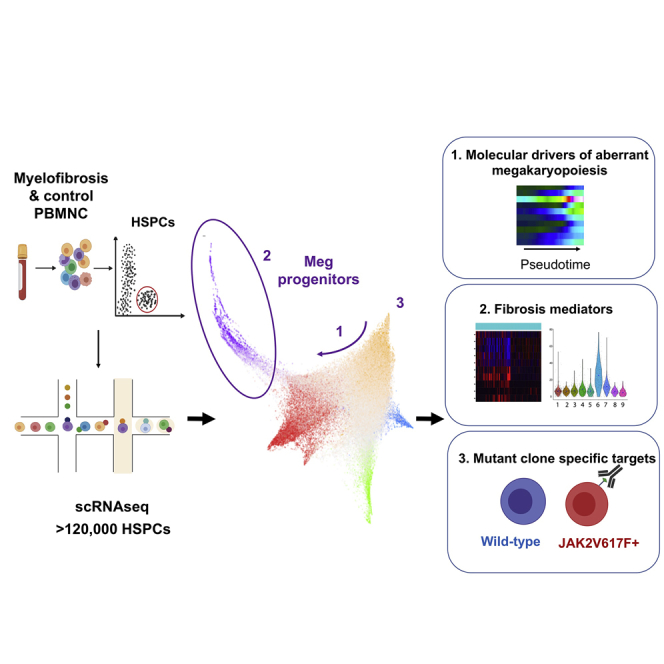

## Teaser

Myelofibrosis (MF) is characterized by increased numbers of morphologically abnormal megakaryocytes (Mks). Single-cell RNA sequencing of >120,000 hematopoietic stem and progenitor cells demonstrated Mk-biased hematopoiesis across clinical and molecular MF subgroups. Mk progenitors were heterogeneous, with distinct expression of inflammatory mediators. Aberrant surface G6B expression on MF stem and progenitors could allow selective immunotherapeutic targeting of the MF clone.

## Introduction

Advances in single-cell technologies have recently provided new insights into the cellular and molecular diversity and pathological mechanisms underlying many diseases, including cancers, pre-malignant and non-malignant conditions (Baslan and Hicks, 2017, Owen et al., 2018, Parikh et al., 2019). Parallel interrogation of mutation status and the transcriptome at a single-cell level provides an unprecedented opportunity to identify cancer-cell-specific targets (Giustacchini et al., 2017, Nam et al., 2019, Rodriguez-Meira et al., 2019). Single-cell resolution also uniquely enables the identification of rare cell types and analysis of combinatorial patterns of gene expression, both of which are necessary to reconstruct differentiation trajectories and to accurately define cellular heterogeneity between populations, such as normal and malignant tissues, as well as to identify the mediators of interactions between different cell types. For example, pathological fibrosis underlies many prevalent diseases, including cancer, where fibrosis is well recognized to be important for disease progression and metastasis (Chandler et al., 2019, Cox and Erler, 2014). It is broadly proposed that pro-fibrotic mediators secreted by cancer cells and infiltrating immune cells activate non-malignant stromal cells, such as myofibroblasts, to deposit collagen fibrosis (Cox and Erler, 2014). However, an understanding of the specific cellular populations that mediate fibrosis in a given disease model, their molecular features, and the cellular pathways through which they are generated is necessary for these cells to be therapeutically targeted.

Myelofibrosis is the most severe of the “myeloproliferative neoplasms” (MPNs), a group of heterogeneous disorders that result from somatic mutations in hematopoietic stem and progenitor cells (HSPCs) affecting Janus Kinase (JAK) signaling. The most common driver mutation is *JAK2V617F*, occurring in ∼60% of myelofibrosis patients (James et al., 2005), with mutations affecting calreticulin (mut*CALR*) found in the majority of other patients (Klampfl et al., 2013, Nangalia et al., 2013). Myelofibrosis can occur as a primary disorder (PMF) or develop secondary to the other MPNs polycythemia vera (post-polycythemia vera myelofibrosis [PPV-MF]) or essential thrombocythemia (post-essential thrombocythaemia myelofibrosis [PET-MF]). Myelofibrosis is characterized by a progressive bone marrow fibrosis that destroys the hematopoietic microenvironment, resulting in the cardinal disease features of cytopenias, mobilization of HSPCs to peripheral blood, extramedullary hematopoiesis, and a high propensity for leukemia. Survival is typically 5–10 years from diagnosis and is not substantially improved by currently available drug therapies (O’Sullivan and Harrison, 2018). Megakaryocytes, the platelet-producing cells in the bone marrow, are dramatically increased in number in myelofibrosis and are one of the key cellular drivers of the destructive bone marrow remodelling by releasing excess pro-fibrotic cytokines and growth factors (Ciurea et al., 2007, Eliades et al., 2011, Martyré et al., 1997, Wen et al., 2015). In normal hematopoiesis, megakaryocyte progenitors (MkPs) have a low proliferation rate, typically undergoing less than 8 cell divisions before mitotic arrest and the onset of polyploidization (Paulus et al., 2004), and megakaryoyctes are relatively rare cells in healthy bone marrow.

The cellular and molecular pathways that give rise to the dramatically increased megakaryocyte numbers and megakaryocyte dysfunction leading to tissue fibrosis are unclear. In traditional models of normal hematopoiesis, megakaryocytes are said to arise from a bipotent progenitor shared with the erythroid (red cell) lineage—the megakaryocyte-erythroid progenitor (MEP) (Akashi et al., 2000, Debili et al., 1996, Kondo et al., 1997, Manz et al., 2002, Pang et al., 2005, Psaila et al., 2016, Psaila and Mead, 2019, Sanada et al., 2016). Recent advances in single-cell technologies, including single-cell transplantation and lineage tracing studies of unperturbed hematopoiesis, have revealed that hematopoiesis occurs over a continuum rather than by distinct, oligopotent intermediate steps (Laurenti and Göttgens, 2018, Psaila and Mead, 2019, Velten et al., 2017) and also that a proportion of hematopoietic stem cells (HSCs), at least in the murine system, are megakaryocyte-biased but retain the capacity for multilineage reconstitution (Adolfsson et al., 2005, Benz et al., 2012, Carrelha et al., 2018, Rodriguez-Fraticelli et al., 2018, Sanjuan-Pla et al., 2013, Shin et al., 2014). Lineage-committed megakaryocytes arising directly from HSCs, sometimes without cell division, have also been reported (Notta et al., 2016, Roch et al., 2015).

Targeting megakaryocytes in myelofibrosis has been shown to ameliorate the disease in mouse models and early-phase human studies (Eliades et al., 2011, Wen et al., 2015), but technical challenges have precluded the extensive study of megakaryopoiesis in myelofibrosis patients. These challenges include the rarity of megakaryocytes in healthy bone marrow, gaps in our knowledge of the cellular pathways of megakaryopoiesis, and their extreme cell size and fragility. In addition, the severe fibrosis typically prevents bone marrow aspiration (“dry tap” aspirate). However, bone marrow HSPCs are mobilized to the peripheral blood in myelofibrosis. In this study, we used this phenomenon to capture peripheral blood HSPCs and perform the first in-depth single-cell analysis of abnormal megakaryocyte differentiation and function in patients with myelofibrosis, suggesting key cellular and molecular targets. Using multiparameter immunophenotyping, functional studies, high-throughput single-cell RNA sequencing (scRNA-seq), targeted single-cell mutational analysis with simultaneous scRNA-seq (TARGET-seq) (Rodriguez-Meira et al., 2019), and single-cell proteomics, we identify potential targets for the inhibition of pathological megakaryocyte differentiation and megakaryocyte-induced fibrosis and validate G6B as a cell surface marker that may enable specific ablation of myelofibrosis cells using immunotherapy. This study illustrates the power of single-cell “multi-omics” in the characterization of cellular heterogeneity in cancers associated with aberrant fibrosis, including the identification of potential therapeutic pathways and cancer-cell-specific targets.

## Results

### Analysis of Mobilized HSPCs Demonstrates Megakaryocyte-Biased HSCs in Myelofibrosis

**Figure 1:**
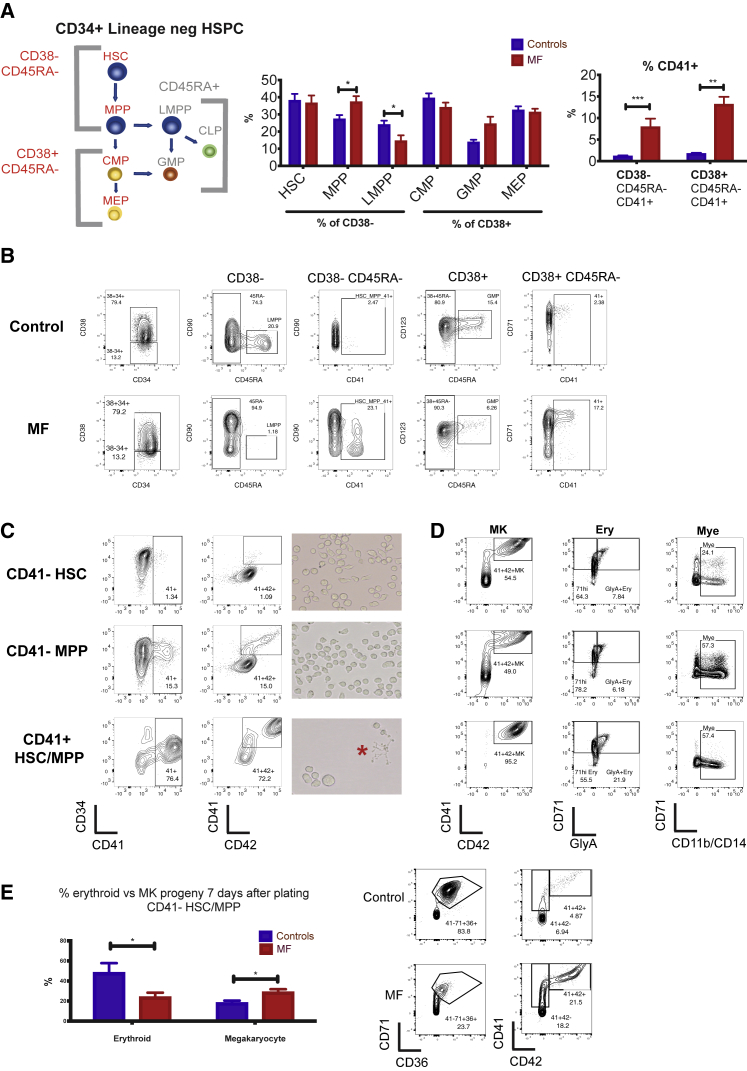
Multipotent Myelofibrosis Hematopoietic Stem and Progenitor Cells (HSPCs) Are Biased for Megakaryocyte Differentiation (A) Left: model of classically defined CD34^+^ lin^−^ HSPC subpopulations, in which multi-potent cells (HSCs, hematopoietic stem cells; MPPs, multi-potent progenitor cells; LMPPs, lymphoid-primed multi-potent progenitors) are CD38^−^ and down-stream progenitors (CMPs, common myeloid progenitors; MEPs, megakaryocyte-erythroid progenitors; GMPs, granulocyte-monocyte progenitors) are CD38^+^. CD45RA^+^ populations (LMPP/GMP) do not have erythroid or megakaryocyte potential. Middle: % of each classically defined HSPC population in the CD34^+^ lin^−^ compartment, demonstrating increased MPPs and reduced LMPPs in myelofibrosis (MF) compared to controls. Right: % cells expressing CD41, a surface antigen previously shown to identify cells with increased potential for megakaryocyte differentiation, is increased in both CD38^−^ CD45RA^−^ (HSC/MPP) and CD38^+^ CD45RA^−^ (CMP/MEP) compartments in myelofibrosis (MF patients, N = 23; controls, N = 14, see also Table S1). (B) Representative FACS plot of a healthy donor control and myelofibrosis patient showing gating strategies. (C) Left: FACS analysis of CD41^−^ HSC (top), CD41^−^ MPP (middle), and CD41^+^ HSC/MPP (bottom) from healthy donors cultured in megakaryocyte differentiation media (with added recombinant human TPO and stem cell factor [SCF]). CD41^+^ HSC/MPP demonstrate increased potential for megakaryocyte differentiation, with faster acquisition of the mature megakaryocyte antigen CD42 at an early time point (day 6). Right: images of cultures showing enlarged cell size and proplatelet formation (red star) indicative of accelerated megakaryocyte differentiation from CD41^+^ HSC/MPP. Representative examples of 3 replicate experiments shown. (D) FACS analysis of CD41^−^ HSC, CD41^−^ MPP and CD41^+^ HSC/MPP from healthy donors cultured for 12–14 days in megakaryocyte (MK), erythroid (Ery), or myeloid (Mye) differentiation media. CD41^+^ HSC/MPP showed a higher % of mature CD41^+^42^+^ megakaryocytes and glycophorin A^+^ CD71^+^ erythroblasts and equivalent CD11b/CD14^+^ myeloid cells versus CD41^−^ fractions. Representative examples of 3 replicate experiments shown. % of total live (7AAD-), single cells shown. (E) Summary chart (left) and representative FACS plots (right) showing percentage of myelofibrosis and control CD41^−^ HSC/MPP cultured in “bi-potent” erythroid and megakaryocyte differentiation media that give rise to megakaryocyte versus erythroid progeny 6 days after plating (gated on live cells). (controls, n = 7; myelofibrosis [MF], n = 8). Charts show mean + SEM,^∗∗∗^p < 0.001; ^∗∗^p ≤ 0.01; ^∗^p < 0.05). See also Figure S1.

Multi-parameter flow cytometric analysis of the CD34^+^ lineage (lin)^−^ HSPC compartment in peripheral blood samples from healthy mobilized apheresis donors and patients with myelofibrosis (Table S1) was performed to compare frequencies of the classically defined HSPC subsets (Figure 1A). This demonstrated reduced lymphoid-primed multipotent progenitors (LMPPs) and increased multi-potent progenitors (MPPs; Figure 1A) in myelofibrosis patients. The cell-surface antigen CD41 has previously been reported to identify cells primed for megakaryocyte differentiation (Gekas and Graf, 2013, Haas et al., 2015, Psaila et al., 2016, Yamamoto et al., 2013). A 5-fold increase in the percentage of CD41^+^ cells was detected within both CD38-negative, early stem/progenitor (HSC and MPP) and CD38-positive, down-stream progenitor (MEP and common myeloid progenitor [CMP]) cell fractions (Figures 1A and 1B), suggesting a bias toward megakaryocyte differentiation originating during the earliest phases of HSC lineage commitment. Morphological analysis of CD38^−^CD41^+^ and CD38^+^CD41^+^ cells from the CD34^+^lin^−^CD45RA^−^ compartment showed undifferentiated blast cell morphology and not mature megakaryocytes (Figure S1A).

The CD41^+^ fraction of human CD38-positive CD34^+^ lin^−^ CD45RA^−^ HSPCs contains megakaryocyte-biased progenitors with significant erythroid differentiation potential as well as unipotent MkP (Miyawaki et al., 2017, Psaila et al., 2016). However, the phenotype of CD41^+^ cells within the CD38-negative HSC/MPP compartment has not previously been defined. We, therefore, sought to determine whether the CD41^+^ HSCs and MPP cells isolated from healthy donors retained a capacity for multi-lineage differentiation or were lineage-committed MkP. CD34^+^ Lin^–^ CD38^–^ CD45RA^–^ CD90^+^ CD41^–^ (CD41^–^ HSC), CD34^+^ Lin^–^ CD38^–^ CD45RA^–^ CD90^–^ CD41^–^ (CD41^–^MPP), and CD34^+^Lin^–^ CD38^–^ CD45RA^–^CD41^+^ (CD41^+^HSC/MPP) cells were isolated by fluorescence-activated cell sorting (FACS) for liquid culture differentiation assays. When stimulated with thrombopoietic cytokines, CD41^+^ HSC/MPP cells showed accelerated megakaryocyte differentiation with a substantially higher proportion of cells expressing the mature megakaryocyte surface antigen CD42, a large cell size, and proplatelet extensions at early time points as compared to CD41^–^ HSCs and MPPs (Figure 1C). In parallel megakaryocyte, erythroid, and myeloid differentiation assays, CD41^+^ HSC/MPP showed a similar potential for CD11b/CD14^+^ myeloid differentiation and a superior potential for CD71^+^/glycophorin A erythroid differentiation than CD41^–^ fractions (Figure 1D).

In comparison to those from healthy donors, CD41^–^ HSC/MPP cells from myelofibrosis patients showed a megakaryocyte versus erythroid differentiation bias (Figure 1E), in keeping with the clinical phenotype of myelofibrosis patients in which excessive megakaryocyte numbers occur in parallel with anemia. In single-cell clonogenic assays supportive of myeloid and erythroid (but not megakaryocytic) colony formation (methocult), CD41^+^ and CD41^–^ fractions of HSCs and MPPs gave rise to expected colony frequencies with myelofibrosis CD41- HSC/MPP showing a bias toward myeloid versus erythroid colonies (Figure S1B). Together, these results support that in myelofibrosis, HSPCs are biased toward megakaryocyte-lineage differentiation from the earliest stem cell compartment, even before expression of canonical megakaryocytic markers.

### High-Throughput scRNA-Seq Identifies a Distinct Pathway for Megakaryocyte Differentiation in Myelofibrosis

To identify the cellular and molecular basis for megakaryocyte-biased hematopoiesis in myelofibrosis without bias from pre-selected cell surface antigens, high-throughput scRNA-seq was performed on 135,929 individual CD34^+^ lin^–^ HSPCs from patients with *JAK2V617F*+ or mut*CALR*+ myelofibrosis (93,157 cells, n = 15) according to World Health Organization (WHO) criteria (Arber et al., 2016) and age-matched healthy donors (42,772 cells, n = 6) by using the 10x Genomics Chromium platform (Table S2). Filtering, quality control and doublet exclusion was performed (Table S3). Healthy donor control and myelofibrosis cells were aggregated and individual donor effect was regressed out, following which no batch effect remained (Figure S1C). A contaminating population of plasmacytic dendritic cells was identified and removed from all down-stream analysis (Figure S1D). Following these steps, 120,196 cells (82,255 myelofibrosis and 37,941 control cells) were used for down-stream analyses (Table S3).

**Figure 2:**
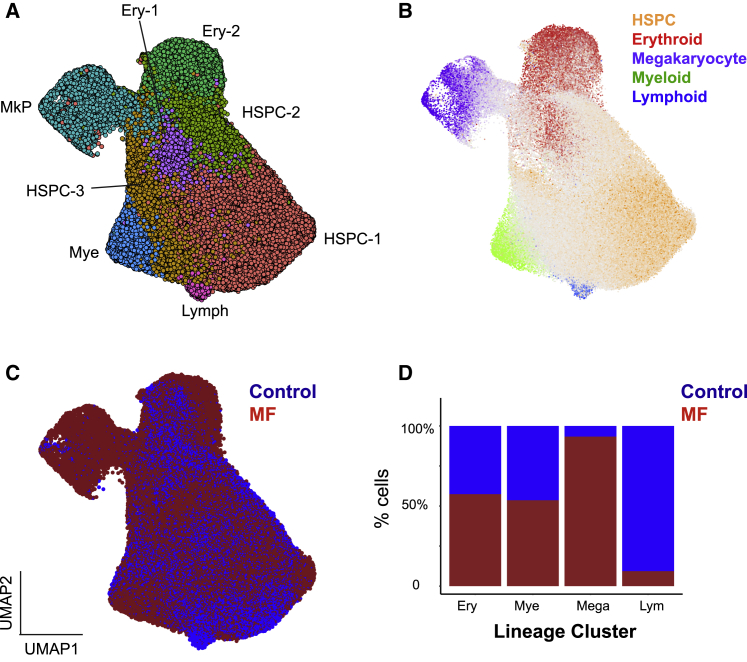
High-Throughput Single-Cell RNA Sequencing of 120,196 CD34^+^ lin^−^ HSPCs from 21 Donors Reveals Marked Expansion of Megakaryocyte Progenitors (MkPs) in Myelofibrosis (A) Dimensionality reduction using UMAP of an aggregate of all control (n = 37,941) and myelofibrosis (n = 82,255) cells identified 8 distinct clusters. Cells were partitioned using the Louvain community-detection clustering method and annotated according to expression of lineage signature genes for hematopoietic cell types (see also Table S4). Abbreviations: Ery - erythroid; Mye - myeloid; Lymph -lymphoid progenitor. (B) Expression of lineage signature gene sets were superimposed on the UMAP (gray, uncommitted or expression of >1 lineage gene set; see also Table S5). (C) Cells were colored according to the donor type (healthy donors, blue; myelofibrosis, red). (D) Myelofibrosis cells were down-sampled to match the number of control cells (37,941 cells). Bar chart shows the % of cells within each annotated lineage progenitor cluster deriving from each donor type. N = 15 for myelofibrosis patients (3 *mutCALR*+ and 12 *JAK2V617F*+) and N = 6 for age-matched controls. See also Figure S1F and Tables S2 and S3.

Dimensionality reduction and unsupervised clustering were performed using a uniform manifold approximation and projection (UMAP) method combined with the Louvain community-detection clustering method to enable identification of distinct cell populations while preserving inter-cluster relationships (Becht et al., 2018) (Figure 2A). A total of 8 clusters were identified and manually annotated by correlation of differentially expressed genes for each cluster with reference marker genes for each lineage (Buenrostro et al., 2018, Hua et al., 2019, Pellin et al., 2019, Popescu et al., 2019) (Figure 2A; Figure S1E; Table S4). “Lineage signature” gene sets were established by an analysis of published datasets (Buenrostro et al., 2018, Hua et al., 2019, Pellin et al., 2019, Popescu et al., 2019) to identify genes selectively expressed in erythroid, myeloid, lymphoid, and megakaryocyte lineage progenitors and uncommitted HSPCs and were superimposed on the UMAPs (Figure 2B; Figures S1F and S2; Table S5).

Most HSPCs expressing megakaryocyte signature genes were derived from myelofibrosis patients with very few from healthy donors (Figures 2B–2D; Figure S1F). The average proportion of HSPCs with a megakaryocyte gene signature was ∼5% for individual myelofibrosis patients, 11-fold higher than for healthy donors (4.98% [range, 0.52%–19.81%] for myelofibrosis versus 0.44% [range, 0%–1.51%] for controls, p < 0.05). Down-sampling the myelofibrosis cells to create a dataset with equal numbers of control and myelofibrosis HSPCs confirmed that over 93% of HSPCs in the MkP cluster originated from myelofibrosis donors, whereas less than 10% lymphoid progenitor cells were from myelofibrosis patients. The fractions of myeloid and erythroid progenitor cells were not significantly different (Figures 2D and S1F), supporting a strong megakaryocyte bias and reduction in lymphoid differentiation in myelofibrosis.

**Figure 3:**
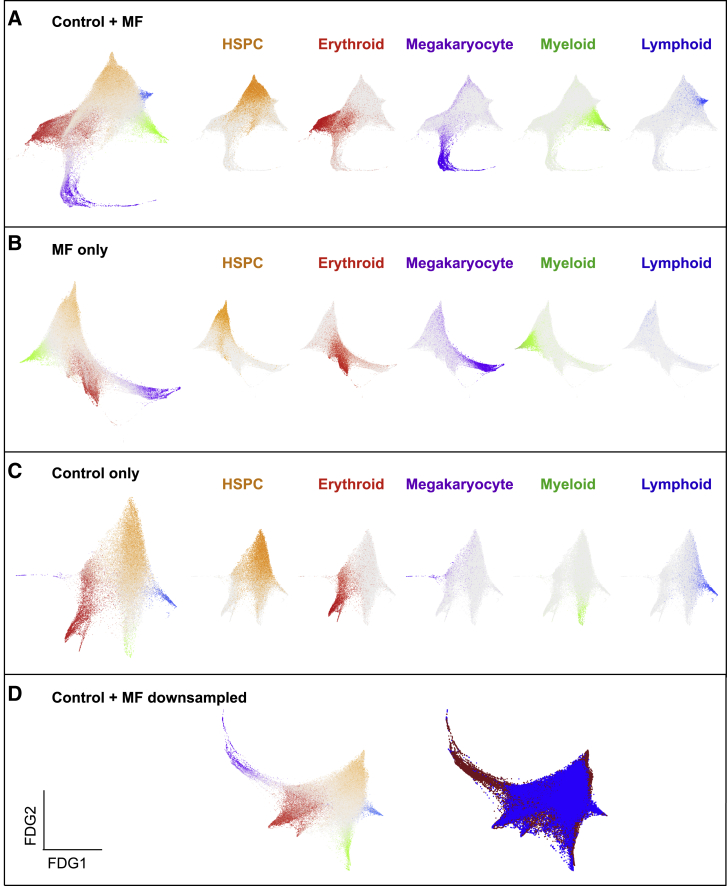
A Distinct Trajectory for Megakaryocyte Differentiation Is Dramatically Expanded in Myelofibrosis (A–D) Force-directed graphs (FDGs) for aggregate of all control + myelofibrosis cells (A), myelofibrosis only (B), control only (C), and control + down-sampled myelofibrosis dataset (D). In (D), the left graph shows lineage signature gene score and in the right graph cells are colored according to the donor type (healthy donors, blue; myelofibrosis, red). Gene expression trajectories are visualized by superimposing the expression scores of lineage signature gene sets on FDG. Grey cells represent uncommitted HSPCs or cells with expression of more than 1 lineage signature. See also Figures S2 and S3 and Table S5.

To study differentiation trajectories, cells were ordered in gene expression space by using force-directed graphs (FDG), and the lineage signature gene scores were superimposed on the graphs (Figures 3A–3D). The HSPC signature highlighted cells at the origin of the trajectory, and erythroid, megakaryocyte, myeloid, and lymphoid trajectories formed distinct paths, with MPP cells primarily located in intermediate positions in the trajectories (gray cells, Figures 3A–3D, left plots; Figure S3). Expression of megakaryocyte signature genes (purple) was observed along a prominent distinct trajectory originating from the apex of the HSPC cluster in the FDG trajectory in myelofibrosis patients (Figures 3B and 3D). In contrast, in healthy donors, very few MkP cells were observed in a distinct differentiation trajectory (Figures 3C and 3D). Notably, most of these cells derived from two of the six healthy donors (donors ID06 and ID09; Figure S3C). Together with functional data (Figure 1), these data support a model in which a direct route for MkP production from HSPCs is aberrantly expanded in JAK2V617F and mut*CALR*-driven myelofibrosis. Our observations were consistent across all clinical and molecular patient subgroups (Figures S3A–S3C), and trajectory analyses using diffusion maps created with Scanpy, an alternative toolkit (Wolf et al., 2018), confirmed findings with our in-house analysis pipeline (Figure S3D).

### Identifying Molecular Drivers for Aberrant Megakaryopoiesis in Myelofibrosis

**Figure 4:**
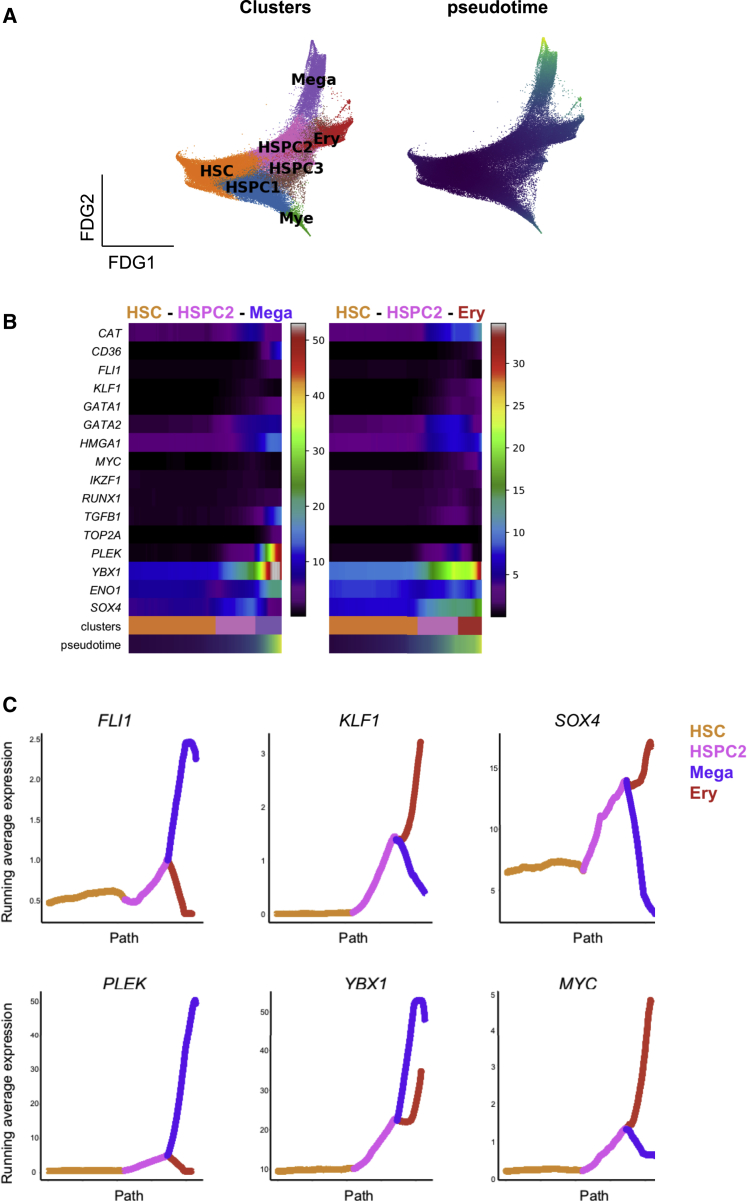
Molecular Regulators That May Drive Aberrant Megakaryocyte Differentiation in Myelofibrosis (A) Left: FDG generated using Scanpy of all myelofibrosis CD34^+^ lin^−^ cells, showing unsupervised clusters based on Louvain community-detection method. Right: pseudotime for the differentiation path from HSCs superimposed on the FDG plot. (B) Expression of selected transcription factor genes over pseudotime from HSC → HSPC2 → megakaryocyte and HSC → HSPC2 → Ery differentiation paths. (C) Expression of 6 genes that are differentially expressed between the erythroid and megakaryocyte trajectories over pseudotime.

We next sought to identify molecular regulators that might specifically drive aberrant megakaryocyte differentiation in myelofibrosis and may potentially be targeted without major toxicity to the other blood cell lineages. Trajectory analysis of all myelofibrosis HSPCs performed using the Scanpy toolkit (Wolf et al., 2018) demonstrated a distinct trajectory for megakaryocyte differentiation through “pseudotime” from an HSC origin (HSC → HSPC2 → Mega; Figure 4A). Expression patterns of 1,639 human transcription factors (Lambert et al., 2018) were analyzed, and transcription factors showing progressive changes over the megakaryocyte and erythroid differentiation trajectories, either increased or decreased expression, were identified. Expected differential expression patterns of transcription factors known to be involved in megakaryocyte versus erythroid lineage specification were observed, e.g., progressive increase in *GATA1* and *GATA2* and antagonistic expression of two key regulators of megakaryocyte-erythroid cell fate decision, namely *FLI1* and *KLF1* (Bouilloux et al., 2008, Doré and Crispino, 2011, Frontelo et al., 2007, Palii et al., 2019, Siripin et al., 2015) (Figures 4B and 4C). Additional genes not previously implicated as regulators of megakaryocyte versus erythroid differentiation showed striking differential expression between the erythroid and megakaryocyte trajectories, including *YBX1*, *HMGA1*, *PLEK*, *SOX4*, and *MYC* (Figures 4B and 4C), suggesting additional targets for strategies to specifically inhibit pathological megakaryopoiesis while preserving erythropoiesis in myelofibrosis patients.

### Identifying Mediators of Megakaryocyte-Induced Fibrosis

**Figure 5:**
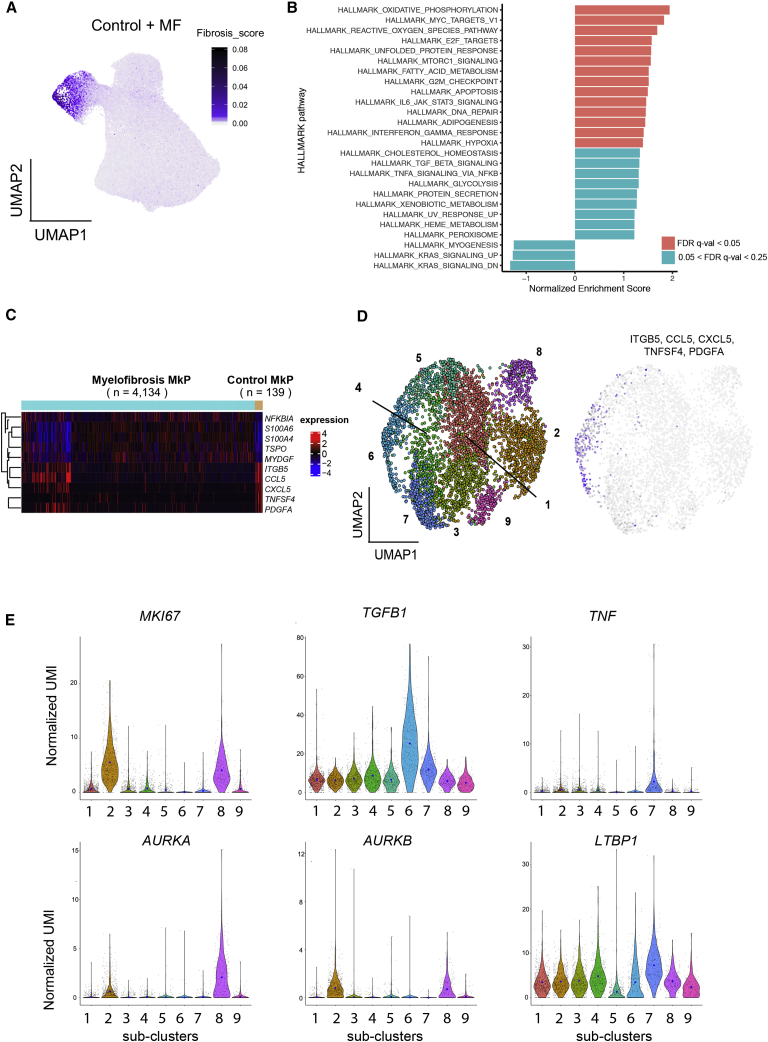
Myelofibrosis MkPs Strongly Express Mediators of Tissue Fibrosis (A) Expression of a 14-gene “fibrosis score” (Table S5) derived from previously published datasets examining bone marrow, liver, and lung fibrosis superimposed on the UMAP of all HSPCs identifies cells in the MkP cluster as the strongest expressers of mediators of tissue fibrosis. (B) HALLMARK pathways from gene set enrichment analysis (GSEA) of all genes pre-ranked according to differential expression in myelofibrosis versus healthy donor MkP. Pathways with a false discovery rate (FDR) q-value of <0.25 are shown. (C) Heatmap showing 10 selected genes differentially expressed between myelofibrosis and control MkP. (D) Left: 9 distinct clusters of myelofibrosis MkP shown on UMAP. Right: expression of signature genes detected in healthy donor MkP and shown in (C) (*ITGB5*, *CCL5*, *CXCL5*, *TNFSF4*, and *PDGFA*) shown on UMAP of myelofibrosis MkP indicates that sub-cluster 6 is transcriptionally similar to control MkP. (E) Heterogenous expression of markers of proliferation (*MKI67*), fibrosis (*TGFB1* and *LTBP1*), inflammation (*TNF*), and treatment targets (*AURKA* and *AURKB)* among myelofibrosis MkP sub-clusters. Blue dots on violin plot indicate mean level of expression. See also Figures S4 and S5.

To evaluate the pathological role of the expanded MkPs in driving bone marrow fibrosis, we next examined potential mediators of fibrosis among HSPCs. Fibrosis regulators were identified from previously published datasets studying lung and liver fibrosis as well as bone marrow fibrosis (Allen et al., 2017, Blackman et al., 2013, Corvol et al., 2015, Gu et al., 2009, Mondet et al., 2015, Mushiroda et al., 2008, Noth et al., 2013, Ulveling et al., 2016, Wattacheril et al., 2017, Wright et al., 2011). Genes detected at expression levels over 1 (using log-transformed unique molecular identifier [UMI]) in our HSPC dataset were selected for a “fibrosis signature” gene score (Table S5). Superimposition of this score on the UMAP for all healthy donor and myelofibrosis HSPCs clearly highlighted the MkP cluster cells as being the key regulators of fibrosis among all HSPCs (Figure 5A).

Healthy donor and myelofibrosis MkPs were then extracted for further analyses (Figure S4A). Gene set enrichment analysis (GSEA) of all expressed genes pre-ranked according to their differential expression between myelofibrosis and control MkP showed significant enrichment of metabolic (e.g., oxidative phosphorylation and fatty acid metabolism) and inflammatory pathways (e.g., transforming growth factor α [TNF-α] signaling and interferon gamma response) in myelofibrosis MkPs (Figure 5B). A subset of myelofibrosis MkPs (cluster cluster 6) showed a similar gene expression profile to control MkPs (Figures 5C and 5D). These cells had a high expression of key inflammatory mediators previously implicated in myelofibrosis, including *PDGFA*, *CCL5*, and *CXCL5* (Figures 5C and 5D) (Eliades et al., 2011, Malara et al., 2018, Mascarenhas et al., 2017). However, the majority of myelofibrosis MkPs had a unique gene expression profile with overall upregulation of genes normally expressed at low levels in healthy donor MkPs (Figures 5C and S4B). This finding suggests that megakaryocyte-induced fibrosis in myelofibrosis is due to both expansion of a population of megakaryocytes analogous to those in normal bone marrow as well as the generation of an aberrant population, an observation that would not have been possible without analysis at the single-cell resolution.

### Myelofibrosis MkPs Demonstrate Molecular Heterogeneity with Differential Expression of Proliferation and Fibrosis Genes

To further dissect cellular and molecular heterogeneity among myelofibrosis MkP, unsupervised clustering using Louvain community detection was performed on myelofibrosis MkP. Nine sub-clusters were identified with distinct expression of fibrosis and proliferation-associated genes (Figures 5D, 5E, and S5A). Genes encoding key mediators of fibrosis (*TGFB1*, *TNF*, and *LTBP1*, which encodes a protein that targets the latent form of transforming growth factor beta [TGF-β] and contributes to its activation; Robertson et al., 2015), were most highly expressed in MkP clusters 6–8, whereas MkP clusters 2 and 8 showed the highest expression of the proliferation marker *MKI67* and a G2M gene signature (Figures 5E and S5B). *AURKA* was selectively expressed in two clusters, with particularly high expression in the minor cluster 8 (Figure 5E). This is of interest as *AURKA* is the target for alisertib (MLN8237), which was recently demonstrated to promote megakaryocyte polyploidization and ameliorate the myelofibrosis phenotype in mouse models (Wen et al., 2015), with some efficacy also in patients with myelofibrosis (Gangat et al., 2019).

Normal megakaryocytes have a low proliferation index, and healthy donor MkPs showed low expression of the proliferation marker *MKI67*. By contrast, *MKI67* was strongly expressed in the majority of myelofibrosis MkPs and the MkP cluster showed the highest expression of *MKI67* among all myelofibrosis HSPC clusters (Figure S5C) as well as an enrichment of a G2M checkpoint gene signature (Figure S5B; Table S5), suggesting that increased proliferation of MkPs may contribute to the pathological accumulation of megakaryocytes in myelofibrosis, in addition to megakaryocyte-biased hematopoiesis.

### Identifying Myelofibrosis Clone-Specific Cell Surface Targets

**Figure 6:**
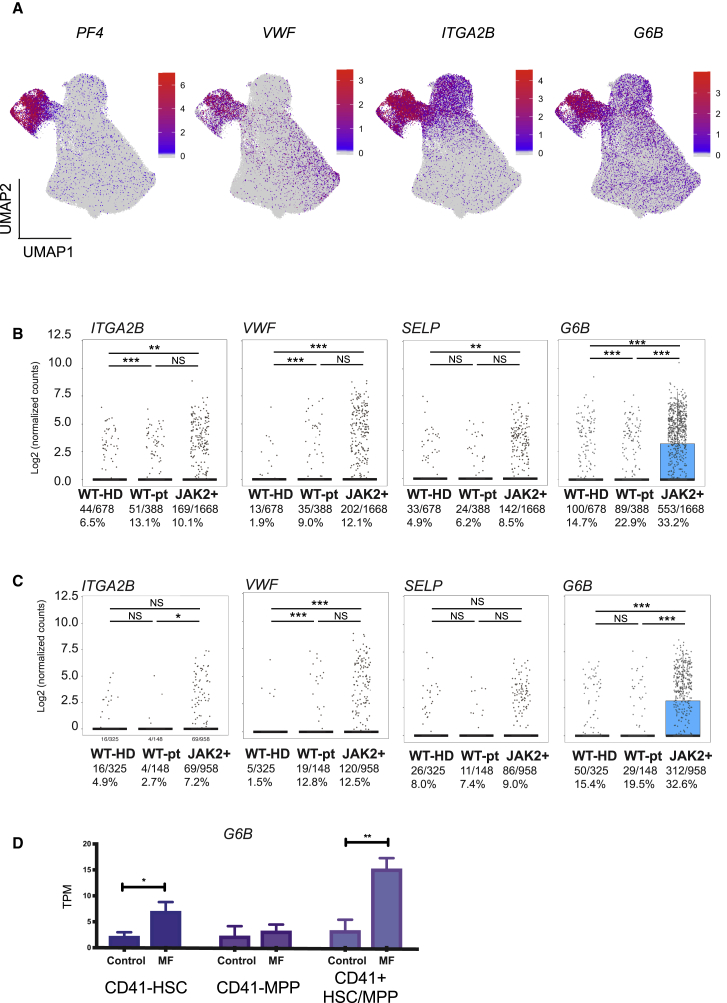
Increased Expression of Megakaryocyte-Associated Genes in Myelofibrosis Is Not Restricted to the MF-MkP Cluster but Is Substantially Higher in Cells Derived from the *JAK2V617F*+ Mutant Clone (A) Expression of intracellular (*PF4* and *VWF*) and cell surface (*ITGA2B* [CD41] and *G6B*) megakaryocyte genes is not limited to myelofibrosis MkPs, particularly for *G6B*. (B and C) Simultaneous targeted mutational profiling and RNA sequencing (TARGET-seq) of 2,734 individual CD34^+^ Lin^−^ HSPCs (B) and CD38-negative stem cells (C) identified by index sorting data show higher expression of megakaryocyte-associated genes *ITGA2B* (CD41), *VWF*, *SELP*, and *G6B* in *JAK2V617F*-mutated (JAK2+) versus wild-type cells from the same patients (WT-pt) or age-matched healthy donor control HSPCs (WT-HD). Fraction and % of cells in which gene expression were detected and are shown. The combined p value for Fisher’s exact test and Wilcoxon rank-sum test is shown (^∗^p < 0.05, ^∗∗^p ≤ 0.01, ^∗∗∗^p ≤ 0.001). Points represent expression values for each single cell, and boxes represent median and quartiles for each group. (D) *G6B* expression in bulk-sorted control and myelofibrosis immunophenotypic HSC (CD34^+^ lin^−^ CD38^−^CD45RA^−^CD90^+^), MPP (CD34^+^ lin^−^ CD38^−^CD45RA^−^CD90^−^), and CD41^+^ HSC/MPP (CD34^+^ lin^−^ CD38-CD45RA^−^ CD41^+^).TPM, transcripts per million. Chart shows mean ± SEM, n = 4 for controls and n = 3 for myelofibrosis; ^∗^p < 0.05; ^∗∗^p ≤ 0.01. See also Figure S6.

Increased expression of megakaryocyte genes in the myelofibrosis aggregate was noted not just within the MkP cluster but also within clusters of uncommitted HSPCs and other lineage-affiliated clusters (Figure 6A). This included intracellular proteins (*PF4* and *VWF*) and also cell surface antigens (*ITGA2B* [CD41] and *MPIG6B* [*G6B*]). Increased expression of *MPIG6B*, encoding the G6B protein, was particularly striking (Figure 6A). G6B is an immunoreceptor tyrosine-based inhibition motif (ITIM)-containing inhibitory receptor, considered to be exclusively expressed on mature megakaryocytes in normal hematopoiesis (Coxon et al., 2017, Senis et al., 2007). As the majority of healthy donor CD34^+^ lin^–^ HSPCs did not express megakaryocyte genes and because mature megakaryocytes normally lose expression of CD34 during differentiation (Tomer, 2004), we hypothesized that aberrant co-expression of stem and progenitor and megakaryocyte surface antigens may enable the selective identification of myelofibrosis clone-derived HSPCs.

Patients with myelofibrosis have distinct genetic subclones of HSPCs, including residual wild-type clones (non-mutated) as well as clones with co-mutations in addition to driver mutations (*JAK2V617F* or mut*CALR*). To determine whether the increase in the expression of megakaryocyte-associated genes was specific to mutant clone-derived HSPCs or due to cell-extrinsic signals affecting both mutated and un-mutated HSPCs, data generated by combined high-sensitivity mutational analysis and parallel transcriptome profiling (TARGET-seq; Rodriguez-Meira et al., 2019) were analyzed. A total of 2,734 cells were examined—678 healthy donor cells plus 2,056 myelofibrosis cells (388 *JAK2* wild type and 1,668 *JAK2V617F* mutated). Expression of megakaryocyte genes, in particular *G6B*, was significantly higher in *JAK2V617F*-mutated HSPCs than in either wild-type cells from the same patients or in wild-type cells from healthy donors (32% versus 22.9% versus 14.7%, respectively; p < 0.001; Figure 6B). Wild-type cells from myelofibrosis patients also showed increased frequency of *G6B* expression, albeit to a lower degree than *JAK2V617F*-positive cells, in keeping with cell-extrinsic signals also contributing to this aberrant megakaryocyte differentiation (Figure 6B).

]The high-throughput TARGET-seq and 10x Chromium datasets included all CD34^+^ lin^–^ cells. Expression levels of G6B were also increased specifically in individual JAK2 mutant CD38^–^ early stem and progenitor cells (HSC/MPP; Figure 6C) identified from the index sorting data of the TARGET-seq cells, and in 100-cell “mini-bulk” preparations of FACS-isolated immunophenotypic CD34^+^ lin^–^ CD38^–^CD45RA^–^ CD90^+^ HSCs, CD34^+^ lin^–^CD38^–^ CD45RA^–^CD90^–^ MPPs, and CD41^+^ HSC/MPPs (Figure 6D).

To examine where cells with specific genotypes fell on the HSPC trajectory, the datasets of myelofibrosis HSPCs analyzed by high-throughput 10x Genomics and TARGET-seq were integrated using Harmony (Korsunsky et al., 2019) (Figure S6A). FDG trajectory analysis showed that both wild-type and mutant progenitors fell in all 3 of the lineage progenitor trajectories (myeloid, erythroid, and megakaryocyte; Figure S6A). However, a higher proportion of the cells in the megakaryocyte and myeloid trajectories were mutant versus wild type than the erythroid trajectory (Figure S6A, right plot). In two patients with 3+ co-mutations in addition to the driver *JAK2V617F* mutation, the increase in *G6B* was observed in all genetic sub-clones detected (Figure S6B).

### Expression of the Cell Surface G6B Protein Selectively Identifies Mutant Clone-Derived HSPCs in Myelofibrosis

**Figure 7:**
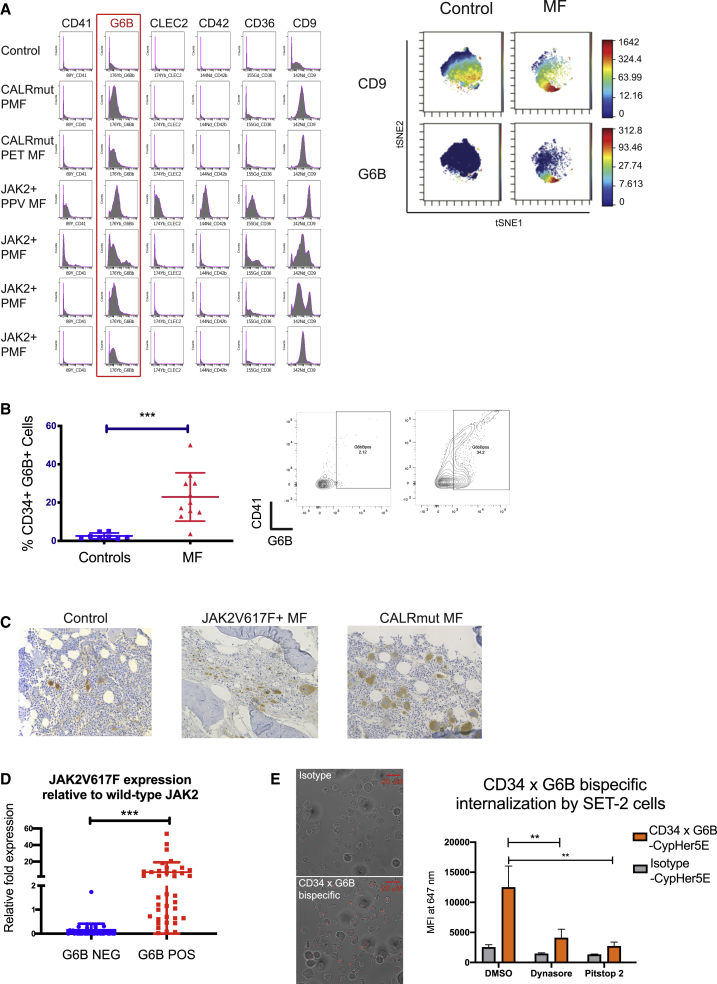
Expression of Cell Surface G6B, a Cell Surface Protein, Identifies Mutant Clone-Derived HSPCs in Myelofibrosis (A) Left: expression of 6 megakaryocyte markers from a panel of 20 HSPC and megakaryocyte cell surface antigens assayed by mass spectrometry time of flight (CyTOF) shows expression of G6B on CD34^+^ HSPCs from patients with primary myelofibrosis (PMF), post-essential thrombocythemia myelofibrosis (PET-MF), and post-polycythaemia vera myelofibrosis (PPV-MF) with either *JAK2V617F* (JAK2+) or calreticulin (mut*CALR*) driver mutations. Histograms show cell count (y axis) by expression level (x axis). Right: viSNE dimensionality reduction plots on a representative control and myelofibrosis sample for CD9 and G6B, illustrating more substantial differential expression of G6B than CD9 in myelofibrosis versus control cells (B) Flow cytometric analysis of G6B expression on CD34^+^ Lin^−^ HSPCs showing significant increase in G6B+ cells in myelofibrosis (% GFP+ cells, 28.8% ± 5.5% versus 2.4% ± 1.0%); chart shows mean + SEM (left) and example plot (right) shown, illustrating expression in both CD41+ and negative cells. ^∗∗^p ≤ 0.01 (t test). Controls (N = 8); myelofibrosis (N = 11). (C) Immunohistochemical staining for G6B (diaminobenzidine, DAB brown) of bone marrow biopsy sections from controls and myelofibrosis patients with *JAK2V617F* and mut*CALR*-positive myelofibrosis showing marked expansion of G6B+ megakaryocytes and progenitors in myelofibrosis. (D) Mononuclear cells from healthy donors and patients with *JAK2V617F*+ myelofibrosis were combined and 50 cell “mini-bulk” replicates were sorted from the G6B+ and G6B− fractions for Taqman qRT-PCR to quantify expression of *JAK2V617F* mutated and wild-type *JAK2*. Chart shows *JAK2V617F* relative to wild-type *JAK2* expression for all mini-bulks from 3 replicate experiments. (E) Internalization of a CD34 × G6B bi-specific antibody and isotype control antibody conjugated to a pH-sensitive cyanine CypHer5E dye that fluoresces at an acidic pH following internalization. Left: representative images show clear intracellular fluorescence for CD34 × G6B bi-specific but not isotype control. Right: mean fluorescence intensity of cells measured by flow cytometry 30 min after addition of antibody with/without two endocytosis inhibitors, Dynasore and Pitstop 2. Data shown using SET-2 cells, chart shows mean + SEM, ** - P < 0.01 n= 3. See also Figure S7.

High-throughput, single-cell proteomics by mass cytometry time of flight (CyTOF) was performed to simultaneously measure 20 surface proteins in multiple samples in parallel by using barcode multiplexing (Figure 7A; Table S7). G6B was consistently detected at substantially higher levels in patients with primary and secondary myelofibrosis and with *JAK2V617F* and *mutCALR* driver mutations than in healthy donors (Figures 7A and 7B). In addition, high cell surface G6B expression was also detected exclusively on *JAK2V617F*-mutated MPN cell lines (HEL and SET2) and not on the other leukemia cell lines, namely K562, HL60, JURKAT, and MARIMO, and HEK (human embryonic kidney) cells (Figure S7A). G6B expression was noted in both the CD41-positive and -negative cell fractions in myelofibrosis by FACS (Figure 7B, right plots).

To examine G6B expression in bone marrow megakaryocytes *in situ*, immunohistochemical staining was performed on trephine biopsy sections from healthy donors and patients with mutCALR and *JAK2V617F*+ myelofibrosis, confirming the expected expression on control megakaryocytes but with a dramatic increase in G6B+ cells in myelofibrosis (Figure 7C).

To confirm G6B as a potential marker of HSPCs derived from the malignant clone, G6B-positive and -negative cells were FACS-isolated from healthy donor and myelofibrosis patient MNCs and the expression of mutant versus wild-type JAK2 was determined by quantitative real-time PCR (Moliterno et al., 2006). Expression of mutant JAK2V617F relative to wild-type JAK2 was dramatically higher in G6B-positive cells (Figure 7D).

To validate that aberrant co-expression of G6B by mutant clone-derived HSPCs may be a potential strategy for immunotherapy, a tool bi-specific antibody was generated to target a stem and progenitor surface antigen (CD34) and G6B and labeled with a pH-sensitive cyanine CypHer5E dye that maximally fluoresces at an acidic pH following cellular internalization. Rapid internalization of the bi-specific by SET-2 cells (a JAK2V617F+ MPN cell line expressing both CD34 and G6B), as indicated by clear intracellular fluorescence, was observed (Figure 7E). Internalization was significantly reduced by two inhibitors of endocytosis, Dynasore (a GTPase inhibitor of dynamin) and Pitstop 2 (an inhibitor of the clathrin terminal domain). Internalization was also observed in CD34-negative, G6B+ HEL cells (Figure 7B) and not in G6B-negative cells nor with an isotype-control antibody, confirming that internalization was mediated by the G6B receptor. This strategy may have the potential to deliver therapeutic antibody conjugates directly to G6B+ mutant clone-derived cells. Together, these data identify G6B as a promising cell surface antigen worthy of further validation as enabling selective targeting of aberrant megakaryocytic differentiation and mutant clone-derived HSPCs in myelofibrosis.

## Discussion

Bone marrow transplant is currently the only potentially curative treatment for myelofibrosis but is associated with significant risk and most patients are ineligible due to age and comorbidities. The introduction of JAK inhibitors has led to significant improvement in symptomatic management, but most patients continue to experience substantial morbidity and a significant reduction in life expectancy. New approaches to treatment are urgently required. Megakaryocytes are well recognized as the key cellular drivers of disease pathogenesis (Malara et al., 2018); however, only one megakaryocyte-targeting therapy—alisertib, a specific inhibitor of aurora kinase—has been developed to date (Gangat et al., 2019, Wen et al., 2015). A major obstacle to identification of novel targets has been the inability to isolate megakaryocytes from patients for detailed study. In the present study, we reasoned that aberrant megakaryopoiesis in myelofibrosis is very likely to be caused by aberrant differentiation of HSPCs, rather than proliferation of mature megakaryocytes alone, and that this process might be amenable to therapeutic targeting to “turn off the supply.” We, therefore, set out to characterize the distinct cellular and molecular features of megakaryocyte differentiation pathways in myelofibrosis by using a combination of single-cell approaches. We demonstrate a dramatic expansion of megakaryocyte differentiation from uncommitted stem and progenitor cells in *JAK2V617F*-driven hematopoiesis. Furthermore, we identify a number of molecular targets that may inhibit the abnormal megakaryocyte differentiation and potentially ablate mutant clone-derived HSPCs and MkPs.

Importantly, several key observations were only possible due to the single-cell-level resolution of study, highlighting the power of single-cell technologies in understanding disease pathology and in novel therapeutic target discovery. First, our data indicate that aberrant megakaryopoiesis in myelofibrosis is due to both a dramatic expansion of MkP with a similar transcriptional signature to healthy donor MkPs, as well as generation of a unique, aberrant MkP population. Second, by simultaneously interrogating the mutational status and the transcriptome of individual cells, we demonstrated that certain megakaryocyte surface antigens, in particular G6B, are markedly over-expressed in mutant clone-derived HSPCs compared with wild-type HSPCs from myelofibrosis patients or healthy donor HSPCs. This validates combinatorial targeting of stem cell (e.g., CD34) and megakaryocyte (e.g., G6B) surface antigens, e.g., with bi-specific antibody therapies as a potential strategy worthy of further investigation for selective ablation of the myelofibrosis clone. As none of the currently available treatments for MPNs reliably induce clonal remissions or substantially reduce fibrosis, this work sets the stage for immunotherapeutic targeting of aberrant hematopoiesis in myelofibrosis. Furthermore, the approach we have adopted and the resulting insights are highly relevant to other studies seeking to identify cancer-cell-specific drug targets and cancer-associated fibrosis in other malignancies, as well as non-malignant disorders of tissue fibrosis.

## STAR★Methods

### Key Resources Table

**Table:** 

REAGENT or RESOURCE	SOURCE	IDENTIFIER
**Antibodies**		

CD34-APC efluor780	eBiosciences (Thermo Fisher Scientific)	Cat#47-0349-42 RRID AB_2573956
CD38-PE-Texas Red	Thermo Fisher Scientific	Cat#MHC03817 RRID AB_10392545
CD90-BV421	BioLegend	Cat#328122 RRID AB_2561420
Lineage antibody cocktail (CD3, CD14, CD16, CD19, CD20, CD56)-BV510	BioLegend	Cat#348807
CD45RA-PE	eBiosciences (Thermo Fisher Scientific)	Cat#12-0458-42 AB_10718395
CD123-PE Cy7	eBiosciences (Thermo Fisher Scientific)	Cat#25-1239-42 RRID AB_1257136
CD41-APC	eBiosciences (Thermo Fisher Scientific)	Cat#17-0419-42 RRID AB_2573144
CD71-AF700	BD	Cat#563769
CD41a-PE	eBiosciences (Thermo Fisher Scientific)	Cat#12-0419-42 RRID AB_10870785
CD42b-APC	eBiosciences (Thermo Fisher Scientific)	Cat#17-0429-42 RRID AB_2573146
CD36-FITC	eBiosciences (Thermo Fisher Scientific)	Cat#11-0369-42 RRID AB_10718972
CD41-PE Cy7	eBiosciences (Thermo Fisher Scientific)	Cat#25-0419-42 RRID AB_2573348
CD42b-PE	BioLegend	Cat#303906 RRID AB_314385
CD11b-APC	eBiosciences (Thermo Fisher Scientific)	Cat#17-0118-42 RRID AB_2016659
CD14-APC	eBiosciences (Thermo Fisher Scientific)	Cat#17-0149-42 RRID AB_10669167
Anti-human G6B antibody (17-4)	Prof. Yotis Senis	N/A
Anti-mouse IgG Alexa Fluor 488 secondary antibody	Thermo Fisher Scientific	Cat#A 10680 RRID AB_2534062
CyTOF antibody cocktail	This paper	See Table S7

**Biological Samples**		

Peripheral blood from patients with myelofibrosis (see Tables S1 and S2)	INForMeD Study (IRAS 199833; REC 16/LO/1376 University of Oxford) Or Hammersmith Hospital Imperial College NHS Trust (R13077; 12275; REC 12/WA/0196)	ID02,ID03,ID04,ID05, ID07,ID08,ID10,ID11, ID12,ID14,ID15,ID16,ID19,ID20,ID21
Peripheral blood from healthy mobilized apheresis donors (see Tables 1 and 2)	INForMeD Study (IRAS 199833; REC 16/LO/1376 University of Oxford) Or Hammersmith Hospital Imperial College NHS Trust (R13077; 12275; REC 12/WA/0196)	ID01,ID06,ID09,ID13, ID17,ID18

**Chemicals, Peptides, and Recombinant Proteins**		

Recombinant human thrombopoietin	PeproTech	Cat#300-18
Recombinant human stem cell Factor	PeproTech	Cat#300-07
Recombinant human erythropoietin	R&D Systems	Cat#287-TC-500
Recombinant human IL3	PeproTech	Cat#200-34
Recombinant human IL6	PeproTech	Cat#200-06
Recombinant human GMCSF	PeproTech	Cat#300-03
Recombinant human G-CSF	PeproTech	Cat#300-23
May Grunewald solution	Sigma-Aldrich Inc. (Merck KGaA)	Cat#63590
Giemsa stain	Sigma-Aldrich Inc. (Merck KGaA)	Cat#48900
Maxpar PBS Buffer	Fluidigm	Cat#201058
Maxpar Cell-ID Cisplatin Viability Stain	Fluidigm	Cat#201064
Maxpar Cell Staining Buffer (CSB)	Fluidigm	Cat#201068
Maxpar Nuclear Antigen Staining Buffer	Fluidigm	Cat#201063
Barcode Perm Buffer	Fluidigm	Cat#201057
Cell-ID 20-Plex Pd Barcoding Kit (Palladium Barcodes)	Fluidigm	Cat#201060
Nuclear Antigen Staining Perm Buffer (NP Buffer)	Fluidigm	Cat#201063
Cell-ID Intercalator-Ir	Fluidigm	Cat#201192A
Maxpar Fix and Perm Buffer	Fluidigm	Cat#201067
EQ Four Element Calibration Beads	Fluidigm	Cat#201078
Cell Conditioning 2 (CC2) antigen retrieval	F. Hoffmann-La Roche Ltd.	Cat#950-123
Ventana DISCOVERY antibody diluent	F. Hoffmann-La Roche Ltd.	Cat#760-108

**Critical Commercial Assays**		

Stemspan SFEM	STEMCELL Technologies	Cat#09650
MethoCult H4435 Enriched	STEMCELL Technologies	Cat#04435
Chromium Single Cell 3′ GEM Library and Gel Bead Kit v2	10x Genomics, Inc.	Cat#1000075
Chromium Single Cell 3′ GEM Library and Gel Bead Kit v3	10x Genomics, Inc.	Cat#1000092
Chromium Chip B Single Cell Kit	10x Genomics, Inc.	Cat#1000074
MiSeq Reagent Kit V2	Illumina	Cat#102-2001
Nextera XT DNA Sample Preparation Kit	Illumina	Cat#FC-131-1024
EasySep Human CD34 Positive Selection Kit	STEMCELL Technologies	Cat#18096
Maxpar X8 Antibody Labeling Kit	Fluidigm	Cat#PRD002
pcDNA™ 3.4 TOPO™ TA Cloning Kit	Thermo Fisher Scientific	A14697
ExpiCHO™ Expression System Kit	Thermo Fisher Scientific	A29133
rProtein A Sepharose 4 Fast Flow Affinity Media	GE HealthCare Life Sciences	17-1279-03
CypHer5E NHS Ester	GE Healthcare Life Sciences, supplied by VWR	VWF PA15401
Monoclonal Anti-DNP antibody, human IgG1 (N297A) isotype control	ACRO Biosystems	DNP-MB273
Microscope slides with 10 flat wells	Hendley-Essex	PH056
Dynasore	Sigma-Aldrich Inc. (Merck KGaA)	D7693
Pitstop2	Sigma-Aldrich Inc. (Merck KGaA)	SML1169

**Deposited Data**		

10X single cell-seq data in this manuscript	This manuscript	GSE144568
TARGET-Seq data	Rodriguez-Meira et al., 2019	GSE122198

**Experimental Models: Cell Lines**		

HEL(human erythroleukemia)	ATCC	RRID:CVCL_8059
JURKAT	ATCC	RRID:CVCL_0367
K562	ATCC	RRID:CVCL_0004
HEK	ATCC	RRID: CVCL_0045
HL-60	ATCC	RRID:CVCL_0002
MARIMO	ATCC	RRID: CVCL_6992
SET-2	Laboratory of Prof. Jacqueline Boultwood	RRID:CVCL_2187

**Oligonucleotides**		

JAK2_WT_VIC sequence VIC TCTCCAC AGACACATAC MGBNFQ	Moliterno et al., 2006	Applied Biosystems Custom Oligo Synthesis service
JAK2V617F_MUT_FAM sequence 6FAM TCCACAGAAACATAC MGBNFQ	Moliterno et al., 2006	Applied Biosystems Custom Oligo Synthesis service
JAK2_FOR AAG CTT TCT CAC AAG CAT TTG GTT T	Moliterno et al., 2006	Eurofins Genomics Custom oligos
JAK2_REV CCA AAT TTT ACA AAC TCC TGA ACC AGA A	Moliterno et al., 2006	Eurofins Genomics Custom oligos

**Software and Algorithms**		

CyTOF Software	Fluidigm	https://www.fluidigm.com/software
Cytobank	Kotecha et al., 2010	https://mrc.cytobank.org
Flowjo version (10.5.3)	FlowJo	https://www.flowjo.com
GraphPad Prism	GraphPad Software Inc.	https://www.graphpad.com
R (v3.6.1)	Team R C, 2013	https://cran.r-project.org/bin/macosx/
Cell Ranger v3.0.1	10x Genomics, Inc.	https://github.com/10XGenomics/cellranger
RStudio (v1.1.463)	Team R S, 2015	https://rstudio.com/products/rstudio/download/
Scanpy (v1.4.5)	Wolf et al., 2018	https://icb-scanpy.readthedocs-hosted.com/en/stable/
TARGET-Seq analysis pipeline	Rodriguez-Meira et al., 2019	https://github.com/albarmeira/TARGET-seq

**Other**		

AUCell_1.6.1	Aibar et al., 2017	https://www.bioconductor.org/packages/release/bioc/vignettes/AUCell/inst/doc/AUCell.html
sva_3.32.1	Johnson et al., 2007	https://bioconductor.org/packages/release/bioc/vignettes/sva/inst/doc/sva.pdf
uwot_0.1.5	McInnes et al., 2018	https://cran.r-project.org/web/packages/uwot/index.html
igraph_1.2.4.2	Csardi and Nepusz, 2005	https://cran.r-project.org/web/packages/igraph/igraph.pdf
RANN_2.6.1	https://cran.r-project.org/web/packages/RANN/index.html	https://github.com/jefferislab/RANN
fa2	Jacomy et al., 2014	https://pypi.org/project/fa2/

### Lead Contact and Materials Availability

Further information and requests for resources or materials will be fulfilled by bethan.psaila@ndcls.ox.ac.uk or adam.mead@imm.ox.ac.uk

#### Cell lines

HEL, JURKAT, K562, HEK, HL60 and MARIMO cells were obtained from the American Type Culture Collection (ATCC). SET-2 cells were kindly provided by Dr. Jacqueline Boultwood and Dr. Andrea Pellagatti (Radcliffe Department of Medicine, University of Oxford). All cells were maintained in culture in RPMI-1630 supplemented with 10% fetal calf serum (FCS) and 1% penicillin-streptomycin. SET-2 cells were supplemented with 20% FCS.

### Experimental Model and Subject Details

A summary of demographic and clinical details of myelofibrosis patients and normal donors used for analysis can be found in Tables S1 and S2.

### Method Details

#### Banking and processing of human samples

Patients and normal donors provided written informed consent in accordance with the Declaration of Helsinki for sample collection, tissue banking and use in research under either the INForMed Study, University of Oxford (IRAS: 199833; REC 16/LO/1376) or Imperial College London (approval reference: R13077; HTA license 12275; REC 12/WA/0196). Cryopreserved peripheral blood mononuclear cells stored in FCS with 10% DMSO were thawed and processed by warming briefly at 37°C, gradual dilution into RPMI-1630 supplemented with 10% FCS and 0.1mg/mL DNase I, centrifuged at 500G for 5 minutes and washed in FACS buffer (PBS + 2mM EDTA + 10% FCS).

#### Fluorescent activated cell sorting (FACS) staining, analysis and cell isolation

FACS-sorting was performed using a Becton Dickinson Aria III or Fusion 2 and cells isolated into 1.5ml Eppendorf tubes or 96-well plates depending on the experiment. Single color stained controls and fluorescence minus one (FMO) controls were used for all experiments. HSPCs were stained with the following antibody cocktail (see Key Resources Table) for 20 minutes at 4°C and passed through a 70 μm mesh cell strainer if necessary prior to sorting: CD34-APC-efluor780; Linege-BV510; CD38-PE-TxRed; CD123-PeCy7; CD45RA-PE; CD71-AF700; CD41-APC; CD90-BV421. The following antibody cocktail was used to analyze cell differentiation: CD34-APC-efluor780, CD71-AF700, CD36-FITC, CD41 PeCy7, CD42 PE, CD11b-APC, CD14-APC. 7AAD was used for live/dead cell exclusion. For G6B immunostaining, cells were stained with anti-human G6B (17-4) for 30 minutes at 4°C (1:100), washed and stained with goat anti-mouse Alexa Fluor 488 secondary IgG antibody (2:200 ThermoFisher Cat#A10680) for 20 minutes in the fridge and washed prior to staining with fluorescence-conjugated commercial antibodies.

#### *In vitro* liquid culture differentiation assays

Cells were isolated by FACS into 1.5 μL eppendorfs, centrifuged at 500G for 5 minutes, resuspended in 100ul culture medium and plated in flat-bottom 96-well plates (Corning). Media used was Stemspan SFEM (StemCell Technologies #09650) + 1% Pen/Strep supplemented with the following cytokines (see also Key Resources)

**Table:** 

Lineage culture	Cytokine	Concentration
Megakaryocyte single lineage	rhTPO	100ng/ml
	rhSCF	50ng/ml
Erythroid single lineage	EPO	1U/ml increasing to 3U/ml from day 6
	IL3	10ng/ml
	IL6	20ng/ml
	SCF	100ng/ml
Myeloid single lineage	SCF	100ng/ml
	G-CSF	20ng/ml
	GM-CSF	20ng/ml
Bi-potent Ery-MK	EPO	1U/ml
	TPO	100ng/ml
	SCF	100ng/ml
	IL3	10ng/ml
	IL6	20ng/ml

Cells were analyzed by FACS on days 6 and 14 (50 μL removed from wells for analysis and replaced with fresh media).

#### Cytospins and MGG

Cells were FACS-isolated into 1.5ml Eppendorf tubes, centrifuged and resuspended into 200 μL PBS and cytospun at 500RPM for 5 minutes onto Superfrost glass slides. May Grunewald Giemsa stain was prepared as per manufacturers protocol, filtered and slides stained in May-Grunewald for 7 minutes followed by 20 minutes in Giemsa then washed in distilled water, air-dried and coverslip applied.

#### Methocult assay

Single cells were FACS-isolated into flat bottomed 96-well plates containing 100 μL of MethoCult™ H4435 Enriched (StemCell Technologies Cat#04435). Colonies were visually inspected and classified 11-14 days after plating. Lineage assignment was made by morphological assessment with verification of ambiguous colonies by plucking and FACS analysis.

#### High-throughput single-cell RNA-sequencing (10x Chromium)

Cells were thawed, stained with FACS antibodies and sorted on a BD Aria III or Fusion 2 as described above and as per recommendations in the 10x Genomics Single Cell Protocols – Cell Preparation Guide. 15,000 CD34+ lineage negative cells were sorted into 2 μL PBS/0.05% BSA (non-acetylated) and then the cell number/volume adjusted to the target for loading onto the 10x Chromium Controller. Samples were processed according to the 10x protocol using the Chromium Single Cell 3′ library and Gel Bead Kits v2 (batch 1) or v3 (batch 2) (10x Genomics). Cells and reagents were prepared and loaded onto the chip and into the Chromium Controller for droplet generation. RT was conducted in the droplets and cDNA recovered through demulsification and bead purification. Pre-amplified cDNA was used for library preparation, multiplexed and sequenced on a HiSeq 2500 (batch 1) or a Novaseq S4 (batch 2) aiming to obtain > 50,000 reads per cell. For some samples, a preliminary, low-depth run was done on a MiSeq using MiSeq Nano Reagent Kit V2 (Illumina Cat#102-2001) to estimate the number of cells and total sequencing required.

#### TARGET-seq analysis

The count matrix for 8 myelofibrosis patients and two healthy donors profiled using 3′TARGET-seq were downloaded from GSE122198, normalized by library size and log2-transformed as previously described (Rodriguez-Meira et al., 2019). Cells were classified into WT-normal (cells from normal donors), WT-patient (non mutant cells present in patient samples) and mutant (cells from patient samples carrying mutations in the genes targeted).

#### RNA sequencing of ‘mini-bulk’ HSPC populations

100 cells from each population were isolated by FACS into 4 μL of lysis buffer containing oligo-dT primer and dNTP mix in 0.2 mL PCR tubes. Cell lysis, RT and PCR preamplification and purification was performed using the Smart-Seq 2 protocol as previously published (Picelli et al., 2014). Libraries were pooled and tagmentation performed using the Illumina Nextera XT DNA sample preparation kit (Illumina Cat #FC-131-1024), libraries pooled and sequenced on a HiSeq 2000.

#### Antibody labeling with metal conjugates and mass cytometry (CyTOF)

Antibodies were purchased pre-conjugated when commercially available. Non-available antibodies were conjugated to lanthanide metals using Maxpar X8 antibody labeling kit according to the manufacturer protocol (version 10). The antibody cocktail used is listed in Table S7. For barcoding and staining, cells were washed with Maxpar PBS buffer (Fluidigm #201058) and stained with 0.5 μM Cell-ID Cisplatin Viability Stain (Fluidigm #201064) in 200 μL Maxpar PBS for 5 mintutes at room temperature for dead cell exclusion. The reaction was quenched with Maxpar Cell Staining Buffer (CSB, Fluidigm #201063) and cells fixed, permeabilized and barcoded using the Cell-ID 20-Plex Pd Barcoding Kit (Fluidigm #201060) as per the manufacturers user guide. Barcoded cells were washed, combined and stained with the antibody cocktail as per Table S7 for 30 minutes at room temperature. Cells were washed with Maxpar Cell Staining Buffer (Fluidigm #201068), fixed in 1.6% formaldehyde, washed and resuspended in Fix&Perm Buffer (Fluidigm Cat#201067) with Cell-ID intercalator-Ir (Fluidigm #201103B) and incubated overnight at 4°C. The following day, cells were washed and analyzed on a Helios (Fluidigm). The mass cytometer was tuned and QC was run prior to acquiring samples according to the manufacturers’ recommendations.

#### G6B Immunohistochemistry

Sections of formalin fixed and paraffin embedded (FFPE) bone marrow trephine biopsies were processed as follows: paraffin was removed, then antigen retrieval was performed using citrate (Roche Cell Conditioning 2 Cat#950-123) pre-treatment for 30 minutes. Sections were washed and incubated with G6B antibody diluted 1:100 in Ventana’s DISCOVERY antibody diluent (Roche Cat#760-108) for 60 minutes at room temperature. Secondary detection was performed using UltraMap DAB anti-Ms HRP detection kit (Roche #760-152) for 16 minutes and slides counterstained with hematoxylin (Roche #760-2021) for 4 minutes and Bluing reagent (Roche #760-2037) for 4 minutes.

#### Sorting G6B+ and G6B- HSPCs for JAK2V617F qRT-PCR

For each experiment, MNCs from myelofibrosis patients and healthy donor controls were thawed and combined 1:1 in FACS buffer prior to antibody staining as described above. 50 G6B+ and G6B- cells were sorted into each well of a 96-well PCR plate (10 replicates per population for each experiment), containing CellsDirect One-Step qRT-PCR kit 2X Reaction Buffer and SuperScript III RT/Platinum Taq Mix (Thermo Fisher Cat#11753100), Ambion SUPERase-In RNase inhibitor (Thermo Fisher Cat#AM2694), TE buffer, JAK2 forward and reverse primers and wild-type and JAK2V617F-specific probe mix (see Key Resources Table). RT and PCR were performed as per manufacturer’s recommendations with 18 pre-amplification cycles and then diluted 5x in TE buffer. Taqman RT-PCR was performed in a 20 μL reaction volume using 4 μL of the diluted cDNA, Taqman Fast Advance Mastermix (Thermo Fisher Cat#4444556) and the primers/probes as detailed in the Key Resources Table. Custom Taqman assays were designed (see Key Resources Table) as previously described (Moliterno et al., 2006) using RT-PCR primers flanking the mutant region plus two Taqman PCR probes specific for the normal or mutant sequence. An Applied Biosystems 7500 Fast Real-Type PCR system was used with the default PCR conditions, with each replicate run in duplicate. Intra-assay replicates varying more than 5% were excluded.

#### CD34 x G6B bispecific antibody generation

The CD34 x G6B bispecific antibody contains a human IgG1 Fc and was produced using ‘knobs-into-holes’ technology, which involves generating a single amino acid substitution in opposite CH3 domains (Ridgway et al., 1996). Each sequence was inserted into the pcDNA 3.4 expression vector (Thermo Fisher). Following preparation of plasmid DNA, each chain was co-transfected into Chinese Hamster Ovarian cells at a 200 mL scale using the ExpiCHO expression system (Thermo Fisher). The Max Titer protocol was followed. Cells were incubated in a 37ᵒC incubator with a humidified atmosphere of 8% CO_2_ in air on an orbital shaker. On the day after transfection, ExpiFectamine CHO Enhancer and ExpiCHO Feed was added to the flask at the appropriate volume and the flask was transferred to a 32ᵒC incubator with a humidified atmosphere of 5% CO_2_ in air on an orbital shaker. On Day 5 post-transfection, the second volume of ExpiCHO Feed was added to the flask. On Day 14, the cells were harvested by centrifuging at 18,000 x g for 30 min. The protein was purified from the supernatant using protein A affinity resin (GE HealthCare Life Sciences). Bound protein was eluted with 20 mM Citrate at pH 2.9 and then immediately neutralized with 10% 1 M Tris. The protein was then further purified using size exclusion chromatography (SEC), and characterized by SDS-PAGE gel and analytical scale SEC.

#### Antibody internalization experiments

The CD34 x G6B bispecific and a non-targeting isotype control (DNP-MB273, Acrobiosystems) were conjugated to CypHer5E (GE Healthcare Life Sciences), a red-excitable, pH-sensitive cyanine dye detected in the APC or Ax647 channel that maximally fluoresces at an acidic pH (i.e., after movement from a receptor on the cell surface to acidic endosomes upon internalization). HEL and SET-2 cells were re-suspended in serum-free, no phenol red RPMI and incubated with DMSO or inhibitors for 30 minutes at 37°C, 5% CO2 prior to the addition of either the CD34 x G6B bispecific or isotype control (5 μg/ml), followed by a further incubation for 30 minutes at 37°C, 5% CO2. For flow cytometry, cells were then washed twice with PBS and re-suspended in PBS for flow analysis on a Beckman Flow CytoFLEX cytometer. For live cell imaging, cells were plated onto slides with flat wells (Hendley-Essex) and imaged on a Zeiss inverted confocal LSM870 with an apochromatic 40X oil immersion objective lens. Two inhibitors were used - Dynasore, a GTPase inhibitor of dynamin (Sigma) (Macia et al., 2006) at 100 μM and Pitstop 2 (Sigma) (von Kleist et al., 2011) at 30 μM concentration, that inhibits the clathrin terminal domain as well as clathrin-independent endocytosis.

#### 10x Genomics single-cell RNA sequencing data pre-processing and integration

Sequencing data in the binary base call (BCL) format were demultiplexed. Unique molecular identifier (UMI) counts were obtained by aligning FASTQ files to the human reference genome (GRCh38 3.0.0) using Cell Ranger software (version 3.0.1) from 10x Genomics. The CellRanger “count” standard pipeline was used to obtain the expression matrix for each individual library for each donor. Cells meeting the following QC parameters (detailed in Table S3) were included in analyses: UMI counts > 1,000 and ≤ maximum UMIs); number of detected genes > 500 and ≤ maximum number of detected genes); the percentage of mitochondrial gene expression < 15% per cell. Genes expressed in at least 10 cells were included. Following application of these filters, 122,154 cells passed quality control (83,753 cells from myelofibrosis patients and 38,401 cells from healthy donors, see Table S3). We scaled UMI counts by normalizing each library size to 10000. The normalized expression values were then log transformed (Ritchie et al., 2015).

#### Dimensionality reduction, removal of individual donor effect and cell clustering

Sparse expression matrices of cells obtaining from the CellRanger output for individual donor were combined. Highly variable genes were identified by fitting mean expression values and the squared coefficient of variation (CV^2^) calculated with a gamma generalized linear model using the “glmgam.fit” function in the statmod package in R as described previously (Brennecke et al., 2013). Using a mean expression value of > 0.05 and the dispersion score of > 0.05, 800 genes were identified as highly variable genes. 12 ribosomal, mitochondrial and heat shock protein genes were removed (Kampinga et al., 2009, Nakao et al., 2004), resulting in 788 highly variable genes being used for down-stream analysis. Donor effect was regressed out using Combat from the sva package (Johnson et al., 2007) by regressing out on the donor IDs. Following this, there was no clear batch effect in the dataset (Figure S1C). Principal Component Analysis (PCA) was then performed on normalized expression values on the first 50 principal components (PCs). An elbow plot was inspected to determine the appropriate number of top PCs capturing the most of variances. Using this approach, we selected the first 20 PCs for further analyses. Uniform Manifold Approximation and Projection (UMAP) analysis was performed using the “uwot” function on the embedded matrix derived from the first 20 PCs and 30 neighbors using cosine as the metric parameter. Cells were clustered using the k-nearest neighbor (KNN) approach, using Euclidean metric as the input parameter. The weighted graph was created with the weight values calculated from the normalized shared number of the nearest neighbors. The function ‘cluster_louvain’ from the igraph package (Csardi and Nepusz, 2005) was then applied to identify clusters based on the weighted graph. Identified clusters were superimposed on the two-dimensional UMAP. We identified a distinct cluster (n = 1,958 cells) expressing a gene signature corresponding to plasmacytoid dendritic cells (pDC) (Figure S1D) and we removed this cluster from further analyses as they are not hematopoietic stem/progenitor cells but contaminating mature cells that fall into the CD34^+^Lin^–^ FACS gate. After removing the pDC cluster, 120,196 cells (82,255 from myelofibrosis donors and 37,941 cells from healthy donors) from all 21 donors (myelofibrosis patients + controls) were included in analyses. Highly variable genes were recalculated, and 817 were identified; 12 of which were removed (heat-shock/mitochondrial/ribosomal genes), resulting in 805 highly variable genes being used for all downstream analyses. Eighteen clusters were initially identified, differentially expressed genes inspected and clusters with similar profiles were merged. This resulted in eight major clusters representing HSPC and lineage progenitor populations, as shown in Figures 2A and 2B.

#### Lineage signature gene sets

Lineage signature gene sets were collated by curating known canonical lineage markers selected from multiple recently published hematopoiesis datasets (Buenrostro et al., 2018, Hua et al., 2019, Pellin et al., 2019, Popescu et al., 2019). Genes selectively expressed in hematopoietic lineage progenitors and uncommitted HSPCs were identified (see Table S5). Expression of each gene was plotted individually on the UMAP plot and genes that were highly expressed and most specifically marked distinct lineage clusters or uncommitted HSPCs in the HSPC UMAP were selected for inclusion in the ‘signature gene sets’ (see Figure S2). These gene sets were used to calculate a ‘lineage gene score’ for each cell, based on the average gene expression of each lineage gene set. These scores were superimposed on the UMAP and FDG plots (Figures 2B and 3).

#### Marker gene identification and cell type annotation

Differentially expressed genes for each cluster were identified using the nonparametric Wilcoxon test on the log-transformed, normalized UMIs to compare expression level. Fisher′s exact test was used to compare the cell frequency expressing each gene as previously described (Giustacchini et al., 2017). *P value*s generated from both tests were then combined using Fisher’s method and were adjusted using the Benjamini-Hochberg (BH) correction. Genes expressed by each individual cluster were compared to all other clusters and differential expression defined as an absolute log2 fold change of ≥ 0.5 and adjusted *P value* of < 0.05, with the fraction of expressing cell frequency of > 0.3. Differentially expressed genes were ranked using *P value*s and log2FC to select up to 50 genes per cluster (Tables S4 and S6). Clusters were identified by manual inspection of differentially expressed genes for canonical marker genes of blood cell lineages. All heatmaps show scaled (z-score) expression values.

#### Down-sampling of myelofibrosis cells

To generate a dataset with equal numbers of myelofibrosis and control cells, myelofibrosis cells were ‘down-sampled’ to 37,941 cells. This number is equal to the maximum number of cells that passed QC in the healthy control. These cells were integrated with all the cells from healthy donors, and analyses from normalization to clustering as described above were repeated. 722 highly variable genes were detected and 12 ribosomal, mitochondrial and heat shock protein genes were removed, resulting in 710 highly variable genes used for subsequent analyses. Seventeen clusters were identified initially, clusters expressing erythroid, megakaryocyte, myeloid and lymphoid genes signatures were merged (Figure S1F). The percentage of cells in each of these lineage clusters from each donor type was quantified (Figure 2D).

#### MkP identification and sub-clustering analysis

Using the same method as described above, cells from the two donor types (healthy donor controls and myelofibrosis patients) were integrated separately. 847 and 680 highly variable genes were detected and 18 and 14 clusters for the MF and control respectively. As shown in Figure S4A, clusters 8, 13, 14, 15, and 16 (n = 8134 cells) were identified as MkP clusters in the myelofibrosis UMAP and clusters 13 and 14 (n = 141 cells) in the healthy donor UMAP. We used the AUCell package (Aibar et al., 2017) to calculate the AUCell score for megakaryocyte signature genes for each individual cell within these clusters. Based on the score distribution, we selected AUCell score > 0.4 (Figure S4A) to define MkP. This resulted in 4,134 and 139 MkP cells being identified from myelofibrosis and healthy control donors respectively. To examine the heterogeneity among myelofibrosis MkP, normalization and clustering were performed as described above. 20 PCs were used for analysis and 1195 highly variable genes were identified after removing 11 ribosomal, mitochondrial, and heatshock protein genes. Clustering analysis revealed 9 sub-clusters of myelofibrosis MkP (Figure 5D).

#### Individual donor analysis

After removing plasmacytoid dendritic cells for each individual, a standard pipeline described above was used. 20 PCs and 30 neighbors were used for UMAP and clustering analyses. 20 PCs and 5 neighbors were used for the force-directed graph (FDG) analysis (Figure S3).

#### Differentiation trajectory analysis

ForceAtlas2 software (Jacomy et al., 2014) was used to visualize differentiation trajectories over the force-directed graph layout. The KNN weighted graph was used as the input for the software, and analysis performed with the following parameters: iterations = 1,000, edgeWeightInfluence = 1, barnesHutTheta = 2, scalingRatio = 1, gravity = 0.05 and jitterTolerance = 1. The output layout was plotted using ggplot2 and superimposed with normalized lineage signature gene scores (average expression values) for myeloid, erythroid, lymphoid and megakaryocyte lineages (Table S5). Transparent factors for selected colors were calculated from normalized expression values across cell types. ggplot2 was used, adding the dynamic alpha parameter values to “geom_point” to control the transparency of colors as shown in Figures 3A, 3B, and 3C.

To identify the differentiation paths, the PAGA function in the Scanpy toolkit was used (Wolf et al., 2018, Wolf et al., 2019). Clusters were identified using canonical lineage maker genes as hematopoietic stem cell (HSC), hematopoietic stem/progenitor cell (HSPC1, HSPC2, HSPC3), erythroid (Ery), megakaryocyte (Mega) and myeloid (Mye) (Figure 4A), with HSC clearly positioned at the root of the trajectory. As shown in Figures 4A and 4B, the predominant path for megakaryocyte differentiation was HSC → HSPC2 → Mk.

Scanpy (version 1.4.5) was used for UMAP, FDG, and diffusion maps analyses (Figure S3D) to compare the results of our in-house analysis pipeline with the existing analysis tool. The ‘sc.pp.combat’ function in scanpy was used to correct donor effect by regression out donor IDs. The ‘sc.pp.highly_variable_genes’ function was used by setting ‘n_top_genes’ to the same number of genes used in the in-house pipeline. 20 PCs and 30 neighbors were used to run UMAP, diffusion map, and FDG functions. Megakaryocyte signature genes (Table S5) were superimposed on the UMAP, diffusion map and FDG.

#### Identifying patters of expression of transcription factor genes along megakaryocyte and erythroid trajectories in myelofibrosis patients

To examine expression of 1,639 transcriptional factors (Lambert et al., 2018) along the megakaryocyte and erythroid differentiation trajectories, the 82,255 myelofibrosis HSPCs were clustered by the Louvain community-detection method (resolution = 0.5). Seven major clusters were identified and projected on the FDG layout using Scanpy. Differentially expressed genes for each cluster were identified as described above. 16 transcription factor genes showing progressive changes, either increased or decreased expression, along the two trajectories were selected (Figures 4B and 4C).

The heatmap was generated by calculating the running average of the gene expression for cells along the path using the function ‘scanpy.pl.paga_path’ in Scanpy to investigate dynamic changes in gene expression. The n.avg parameter was set at 5000 for the number of data points to include in the computation of the running average, to ensure a smooth transition.

#### GeneSet Enrichment Analysis

GSEA was performed using GSEA software (https://www.gsea-msigdb.org/gsea/index.jsp) with ‘Run GSEAPreranked’ and default parameters. The HALLMARK gene set used for the analysis was downloaded from MSigDB (https://www.broadinstitute.org/gsea/msigdb/collections.jsp). 9,313 expressed genes were used for the analysis. Genes were pre-ranked in order of their differential expression between myelofibrosis and healthy donor megakaryocyte progenitors. The Pathways with FDR q-value < 0.25 were selected showing in Figure 5B.

#### Integration of 10x Genomics and TARGET-seq datasets

37,941 cells (the down-sampled dataset) from 15 myelofibrosis patients processed using the Chromium platform (10x Genomics) and 2,071 cells from 8 myelofibrosis patients (obtained from Rodriguez-Meira et al., 2019; GSE122198) were projected into a shared embedding with Harmony (Korsunsky et al., 2019), using the top 20 PCA dimensions. The effect of the platform (10X Genomics/TARGET-seq) and donor were simultaneously accounted for and introduced as covariates. Then, dimensionality reduction and force directed graph analysis were performed as described above (Dimensionality reduction, removal of individual donor effect and cell clustering). To quantify the proportion of mutant and wild-type cells in each lineage progenitor cluster, clusters were identified by inspection of differentially expressed genes and super-imposition of the lineage gene set scores on the UMAP and FDG graphs as described above, and mutant and wild-type cells enumerated for myeloid, eythoid and megakaryocyte progenitor clusters. Proportions of wild-type and mutant cells for each of cluster pair were compared using a Chi-square test (p value) with Yates’ continuity correction.

### Quantification and Statistical Analysis

#### Flow cytometry and CyTOF data analysis

Flow cytometry data was analyzed using FlowJo software (v10.5.3). Summary charts and associated statistical analyses were performed using GraphPad Prism (v8.1.0). Helios CyTOF Software (v6.7) was used for processing of FCS 3.0 files, normalization to EQ Beads, concatenation of multiple files and debarcoding. Data was then analyzed and histograms and viSNE plots generated using CytoBank.org.

Statistical tests used, numbers of replicates and definitions of statistical significance are described in the relevant figure legends. All bar charts show mean ± standard error of the mean and were generated using GraphPad Prism (v.8.1.0).

To compare the proportions of wild-type versus mutant cells in lineage progenitor clusters (Figure S6A), Fisher’s exact test was used. To compare the expression of key genes in different sub-clones within the same patients (Figures 6B, 6C, and S6B), Fisher’s test was used to compare the expression frequencies between the groups and Wilcoxon rank sum test to compare the expression levels. P values were combined using Fisher’s method, and the combined p value for each pairwise comparison are reported in Figure S6.

### Data and Code Availability

10x Genomics single cell RNA-sequencing data has been submitted to GEO database (Accession Number GEO: GSE144568). TARGET-seq single cell RNA-sequencing data is available at Accession Number GEO: GSE122198. The Shiny application for visualization of the data from patients and healthy donors in this study is available at https://github.com/supatt-lab/SingCellaR-myelofibrosis. R scripts used for the analysis are available upon request.

## Supporting Citations

The following reference appears in the Supplemental Information: Passamonti et al., 2010.

## Supplemental Information

Document S1. Figures S1–S7 and Tables S1, S5, and S7

Table S2. Detailed Clinical Information of Patients from Whom Samples Were Studied by High-Throughput Single-Cell RNA Sequencing (10x Chromium), Related to Figures 2, 3, 4, 5, and 6A

Table S3. Quality Control and Cells Filtered Out during Quality-Control Steps on Single-Cell RNA Sequencing Data, Related to Figures 2, 3, 4, 5, and 6A

Table S4. Genes Differentially Expressed in Each of the Louvain Clusters as Shown on the Aggregate UMAP of All 120,196 Control Plus Myelofibrosis Cells (up to 50 Genes Listed Per Cluster), Related to Figure 2 Differentially expressed genes for each cluster (group A) are calculated versus all other cells (group B). Abbreviations: ExpFreqA – number of cells in cluster A expressing given gene; ExpFreqB – number of cells in all other clusters expressing given gene; TotalA / TotalB – total number of cells in group A/B; ExpFraction – Fraction of cells in group A / Group B expressing given gene.

Table S6. Genes Differentially Expressed in Louvain Clusters as Shown on the UMAP of Myelofibrosis Megakaryocyte Progenitor Cells, Related to Figure 5 Differentially expressed genes for each cluster (group A) are calculated versus all other cells (group B). Abbreviations: ExpFreqA – number of cells in cluster A expressing given gene; ExpFreqB – number of cells in all other clusters expressing given gene; TotalA / TotalB – total number of cells in group A/B; ExpFraction – Fraction of cells in group A / Group B expressing given gene.

Document S2. Article plus Supplemental Information

## References

[bib1] Adolfsson J., Månsson R., Buza-Vidas N., Hultquist A., Liuba K., Jensen C.T., Bryder D., Yang L., Borge O.J., Thoren L.A. (2005). Identification of Flt3+ lympho-myeloid stem cells lacking erythro-megakaryocytic potential a revised road map for adult blood lineage commitment. Cell.

[bib2] Aibar S., González-Blas C.B., Moerman T., Huynh-Thu V.A., Imrichova H., Hulselmans G., Rambow F., Marine J.C., Geurts P., Aerts J. (2017). SCENIC: single-cell regulatory network inference and clustering. Nat. Methods.

[bib3] Akashi K., Traver D., Miyamoto T., Weissman I.L. (2000). A clonogenic common myeloid progenitor that gives rise to all myeloid lineages. Nature.

[bib4] Allen R.J., Porte J., Braybrooke R., Flores C., Fingerlin T.E., Oldham J.M., Guillen-Guio B., Ma S.F., Okamoto T., John A.E. (2017). Genetic variants associated with susceptibility to idiopathic pulmonary fibrosis in people of European ancestry: a genome-wide association study. Lancet Respir. Med..

[bib5] Arber D.A., Orazi A., Hasserjian R., Thiele J., Borowitz M.J., Le Beau M.M., Bloomfield C.D., Cazzola M., Vardiman J.W. (2016). The 2016 revision to the World Health Organization classification of myeloid neoplasms and acute leukemia. Blood.

[bib6] Baslan T., Hicks J. (2017). Unravelling biology and shifting paradigms in cancer with single-cell sequencing. Nat. Rev. Cancer.

[bib7] Becht E., McInnes L., Healy J., Dutertre C.A., Kwok I.W.H., Ng L.G., Ginhoux F., Newell E.W. (2018). Dimensionality reduction for visualizing single-cell data using UMAP. Nat. Biotechnol..

[bib8] Benz C., Copley M.R., Kent D.G., Wohrer S., Cortes A., Aghaeepour N., Ma E., Mader H., Rowe K., Day C. (2012). Hematopoietic stem cell subtypes expand differentially during development and display distinct lymphopoietic programs. Cell Stem Cell.

[bib9] Blackman S.M., Commander C.W., Watson C., Arcara K.M., Strug L.J., Stonebraker J.R., Wright F.A., Rommens J.M., Sun L., Pace R.G. (2013). Genetic modifiers of cystic fibrosis-related diabetes. Diabetes.

[bib10] Bouilloux F., Juban G., Cohet N., Buet D., Guyot B., Vainchenker W., Louache F., Morlé F. (2008). EKLF restricts megakaryocytic differentiation at the benefit of erythrocytic differentiation. Blood.

[bib11] Brennecke P., Anders S., Kim J.K., Kołodziejczyk A.A., Zhang X., Proserpio V., Baying B., Benes V., Teichmann S.A., Marioni J.C., Heisler M.G. (2013). Accounting for technical noise in single-cell RNA-seq experiments. Nat. Methods.

[bib12] Buenrostro J.D., Corces M.R., Lareau C.A., Wu B., Schep A.N., Aryee M.J., Majeti R., Chang H.Y., Greenleaf W.J. (2018). Integrated Single-Cell Analysis Maps the Continuous Regulatory Landscape of Human Hematopoietic Differentiation. Cell.

[bib13] Carrelha J., Meng Y., Kettyle L.M., Luis T.C., Norfo R., Alcolea V., Boukarabila H., Grasso F., Gambardella A., Grover A. (2018). Hierarchically related lineage-restricted fates of multipotent haematopoietic stem cells. Nature.

[bib14] Chandler C., Liu T., Buckanovich R., Coffman L.G. (2019). The double edge sword of fibrosis in cancer. Transl. Res..

[bib15] Ciurea S.O., Merchant D., Mahmud N., Ishii T., Zhao Y., Hu W., Bruno E., Barosi G., Xu M., Hoffman R. (2007). Pivotal contributions of megakaryocytes to the biology of idiopathic myelofibrosis. Blood.

[bib16] Corvol H., Blackman S.M., Boëlle P.Y., Gallins P.J., Pace R.G., Stonebraker J.R., Accurso F.J., Clement A., Collaco J.M., Dang H. (2015). Genome-wide association meta-analysis identifies five modifier loci of lung disease severity in cystic fibrosis. Nat. Commun..

[bib17] Cox T.R., Erler J.T. (2014). Molecular pathways: connecting fibrosis and solid tumor metastasis. Clin. Cancer Res..

[bib18] Coxon C.H., Geer M.J., Senis Y.A. (2017). ITIM receptors: more than just inhibitors of platelet activation. Blood.

[bib19] Csardi G., Nepusz T. (2005). The igraph software package for complex network research. InterJournal Complex Systems.

[bib20] Debili N., Coulombel L., Croisille L., Katz A., Guichard J., Breton-Gorius J., Vainchenker W. (1996). Characterization of a bipotent erythro-megakaryocytic progenitor in human bone marrow. Blood.

[bib21] Doré L.C., Crispino J.D. (2011). Transcription factor networks in erythroid cell and megakaryocyte development. Blood.

[bib22] Eliades A., Papadantonakis N., Bhupatiraju A., Burridge K.A., Johnston-Cox H.A., Migliaccio A.R., Crispino J.D., Lucero H.A., Trackman P.C., Ravid K. (2011). Control of megakaryocyte expansion and bone marrow fibrosis by lysyl oxidase. J. Biol. Chem..

[bib23] Frontelo P., Manwani D., Galdass M., Karsunky H., Lohmann F., Gallagher P.G., Bieker J.J. (2007). Novel role for EKLF in megakaryocyte lineage commitment. Blood.

[bib24] Gangat N., Marinaccio C., Swords R., Watts J.M., Gurbuxani S., Rademaker A., Fought A.J., Frankfurt O., Altman J.K., Wen Q.J. (2019). Aurora kinase A inhibition provides clinical benefit, normalizes megakaryocytes and reduces bone marrow fibrosis in patients with myelofibrosis. Clin. Cancer Res..

[bib25] Gekas C., Graf T. (2013). CD41 expression marks myeloid-biased adult hematopoietic stem cells and increases with age. Blood.

[bib26] Giustacchini A., Thongjuea S., Barkas N., Woll P.S., Povinelli B.J., Booth C.A.G., Sopp P., Norfo R., Rodriguez-Meira A., Ashley N. (2017). Single-cell transcriptomics uncovers distinct molecular signatures of stem cells in chronic myeloid leukemia. Nat. Med..

[bib27] Gu Y., Harley I.T., Henderson L.B., Aronow B.J., Vietor I., Huber L.A., Harley J.B., Kilpatrick J.R., Langefeld C.D., Williams A.H. (2009). Identification of IFRD1 as a modifier gene for cystic fibrosis lung disease. Nature.

[bib28] Haas S., Hansson J., Klimmeck D., Loeffler D., Velten L., Uckelmann H., Wurzer S., Prendergast A.M., Schnell A., Hexel K. (2015). Inflammation-Induced Emergency Megakaryopoiesis Driven by Hematopoietic Stem Cell-like Megakaryocyte Progenitors. Cell Stem Cell.

[bib29] Hua P., Roy N., de la Fuente J., Wang G., Thongjuea S., Clark K., Roy A., Psaila B., Ashley N., Harrington Y. (2019). Single-cell analysis of bone marrow-derived CD34+ cells from children with sickle cell disease and thalassemia. Blood.

[bib30] Jacomy M., Venturini T., Heymann S., Bastian M. (2014). ForceAtlas2, a continuous graph layout algorithm for handy network visualization designed for the Gephi software. PLoS One.

[bib31] James C., Ugo V., Le Couédic J.P., Staerk J., Delhommeau F., Lacout C., Garçon L., Raslova H., Berger R., Bennaceur-Griscelli A. (2005). A unique clonal JAK2 mutation leading to constitutive signalling causes polycythaemia vera. Nature.

[bib32] Johnson W.E., Li C., Rabinovic A. (2007). Adjusting batch effects in microarray expression data using empirical Bayes methods. Biostatistics.

[bib33] Kampinga H.H., Hageman J., Vos M.J., Kubota H., Tanguay R.M., Bruford E.A., Cheetham M.E., Chen B., Hightower L.E. (2009). Guidelines for the nomenclature of the human heat shock proteins. Cell Stress Chaperones.

[bib34] Klampfl T., Gisslinger H., Harutyunyan A.S., Nivarthi H., Rumi E., Milosevic J.D., Them N.C., Berg T., Gisslinger B., Pietra D. (2013). Somatic mutations of calreticulin in myeloproliferative neoplasms. N. Engl. J. Med..

[bib35] Kondo M., Weissman I.L., Akashi K. (1997). Identification of clonogenic common lymphoid progenitors in mouse bone marrow. Cell.

[bib36] Korsunsky I., Millard N., Fan J., Slowikowski K., Zhang F., Wei K., Baglaenko Y., Brenner M., Loh P.R., Raychaudhuri S. (2019). Fast, sensitive and accurate integration of single-cell data with Harmony. Nat. Methods.

[bib37] Kotecha N., Krutzik P.O., Irish J.M. (2010). Web-based analysis and publication of flow cytometry. Curr. Protoc. Cytom..

[bib38] Lambert S.A., Jolma A., Campitelli L.F., Das P.K., Yin Y., Albu M., Chen X., Taipale J., Hughes T.R., Weirauch M.T. (2018). The Human Transcription Factors. Cell.

[bib39] Laurenti E., Göttgens B. (2018). From haematopoietic stem cells to complex differentiation landscapes. Nature.

[bib40] Macia E., Ehrlich M., Massol R., Boucrot E., Brunner C., Kirchhausen T. (2006). Dynasore, a cell-permeable inhibitor of dynamin. Dev. Cell.

[bib41] Malara A., Abbonante V., Zingariello M., Migliaccio A., Balduini A. (2018). Megakaryocyte Contribution to Bone Marrow Fibrosis: many Arrows in the Quiver. Mediterr. J. Hematol. Infect. Dis..

[bib42] Manz M.G., Miyamoto T., Akashi K., Weissman I.L. (2002). Prospective isolation of human clonogenic common myeloid progenitors. Proc. Natl. Acad. Sci. USA.

[bib43] Martyré M.C., Le Bousse-Kerdiles M.C., Romquin N., Chevillard S., Praloran V., Demory J.L., Dupriez B. (1997). Elevated levels of basic fibroblast growth factor in megakaryocytes and platelets from patients with idiopathic myelofibrosis. Br. J. Haematol..

[bib44] Mascarenhas J.O., Talpaz M., Gupta V., Foltz L.M., Savona M.R., Paquette R., Turner A.R., Coughlin P., Winton E., Burn T.C. (2017). Primary analysis of a phase II open-label trial of INCB039110, a selective JAK1 inhibitor, in patients with myelofibrosis. Haematologica.

[bib45] McInnes L., Healy J., Melville J. (2018). UMAP: Uniform manifold approximation and projection for dimension reduction. arXiv.

[bib46] Miyawaki K., Iwasaki H., Jiromaru T., Kusumoto H., Yurino A., Sugio T., Uehara Y., Odawara J., Daitoku S., Kunisaki Y. (2017). Identification of unipotent megakaryocyte progenitors in human hematopoiesis. Blood.

[bib47] Moliterno A.R., Williams D.M., Rogers O., Spivak J.L. (2006). Molecular mimicry in the chronic myeloproliferative disorders: reciprocity between quantitative JAK2 V617F and Mpl expression. Blood.

[bib48] Mondet J., Hussein K., Mossuz P. (2015). Circulating Cytokine Levels as Markers of Inflammation in Philadelphia Negative Myeloproliferative Neoplasms: Diagnostic and Prognostic Interest. Mediators Inflamm..

[bib49] Mushiroda T., Wattanapokayakit S., Takahashi A., Nukiwa T., Kudoh S., Ogura T., Taniguchi H., Kubo M., Kamatani N., Nakamura Y., Pirfenidone Clinical Study Group (2008). A genome-wide association study identifies an association of a common variant in TERT with susceptibility to idiopathic pulmonary fibrosis. J. Med. Genet..

[bib50] Nakao A., Yoshihama M., Kenmochi N. (2004). RPG: the Ribosomal Protein Gene database. Nucleic Acids Res..

[bib51] Nam A.S., Kim K.T., Chaligne R., Izzo F., Ang C., Taylor J., Myers R.M., Abu-Zeinah G., Brand R., Omans N.D. (2019). Somatic mutations and cell identity linked by Genotyping of Transcriptomes. Nature.

[bib52] Nangalia J., Massie C.E., Baxter E.J., Nice F.L., Gundem G., Wedge D.C., Avezov E., Li J., Kollmann K., Kent D.G. (2013). Somatic CALR mutations in myeloproliferative neoplasms with nonmutated JAK2. N. Engl. J. Med..

[bib53] Noth I., Zhang Y., Ma S.F., Flores C., Barber M., Huang Y., Broderick S.M., Wade M.S., Hysi P., Scuirba J. (2013). Genetic variants associated with idiopathic pulmonary fibrosis susceptibility and mortality: a genome-wide association study. Lancet Respir. Med..

[bib54] Notta F., Zandi S., Takayama N., Dobson S., Gan O.I., Wilson G., Kaufmann K.B., McLeod J., Laurenti E., Dunant C.F. (2016). Distinct routes of lineage development reshape the human blood hierarchy across ontogeny. Science.

[bib55] O’Sullivan J.M., Harrison C.N. (2018). Myelofibrosis: clinicopathologic features, prognosis, and management. Clin. Adv. Hematol. Oncol..

[bib56] Owen R.P., White M.J., Severson D.T., Braden B., Bailey A., Goldin R., Wang L.M., Ruiz-Puig C., Maynard N.D., Green A. (2018). Single cell RNA-seq reveals profound transcriptional similarity between Barrett’s oesophagus and oesophageal submucosal glands. Nat. Commun..

[bib57] Palii C.G., Cheng Q., Gillespie M.A., Shannon P., Mazurczyk M., Napolitani G., Price N.D., Ranish J.A., Morrissey E., Higgs D.R., Brand M. (2019). Single-Cell Proteomics Reveal that Quantitative Changes in Co-expressed Lineage-Specific Transcription Factors Determine Cell Fate. Cell Stem Cell.

[bib58] Pang L., Weiss M.J., Poncz M. (2005). Megakaryocyte biology and related disorders. J. Clin. Invest..

[bib59] Parikh K., Antanaviciute A., Fawkner-Corbett D., Jagielowicz M., Aulicino A., Lagerholm C., Davis S., Kinchen J., Chen H.H., Alham N.K. (2019). Colonic epithelial cell diversity in health and inflammatory bowel disease. Nature.

[bib60] Passamonti F., Cervantes F., Vannucchi A.M., Morra E., Rumi E., Pereira A., Guglielmelli P., Pungolino E., Caramella M., Maffioli M. (2010). A dynamic prognostic model to predict survival in primary myelofibrosis: a study by the IWG-MRT (International Working Group for Myeloproliferative Neoplasms Research and Treatment). Blood.

[bib61] Paulus J.M., Debili N., Larbret F., Levin J., Vainchenker W. (2004). Thrombopoietin responsiveness reflects the number of doublings undergone by megakaryocyte progenitors. Blood.

[bib62] Pellin D., Loperfido M., Baricordi C., Wolock S.L., Montepeloso A., Weinberg O.K., Biffi A., Klein A.M., Biasco L. (2019). A comprehensive single cell transcriptional landscape of human hematopoietic progenitors. Nat. Commun..

[bib63] Picelli S., Faridani O.R., Björklund A.K., Winberg G., Sagasser S., Sandberg R. (2014). Full-length RNA-seq from single cells using Smart-seq2. Nat. Protoc..

[bib64] Popescu D.M., Botting R.A., Stephenson E., Green K., Webb S., Jardine L., Calderbank E.F., Polanski K., Goh I., Efremova M. (2019). Decoding human fetal liver haematopoiesis. Nature.

[bib65] Psaila B., Mead A.J. (2019). Single-cell approaches reveal novel cellular pathways for megakaryocyte and erythroid differentiation. Blood.

[bib66] Psaila B., Barkas N., Iskander D., Roy A., Anderson S., Ashley N., Caputo V.S., Lichtenberg J., Loaiza S., Bodine D.M. (2016). Single-cell profiling of human megakaryocyte-erythroid progenitors identifies distinct megakaryocyte and erythroid differentiation pathways. Genome Biol..

[bib67] Ridgway J.B., Presta L.G., Carter P. (1996). “Knobs-into-holes” engineering of antibody CH3 domains for heavy chain heterodimerization. Protein Eng..

[bib68] Ritchie M.E., Phipson B., Wu D., Hu Y., Law C.W., Shi W., Smyth G.K. (2015). limma powers differential expression analyses for RNA-sequencing and microarray studies. Nucleic Acids Res..

[bib69] Robertson I.B., Horiguchi M., Zilberberg L., Dabovic B., Hadjiolova K., Rifkin D.B. (2015). Latent TGF-β-binding proteins. Matrix Biol..

[bib70] Roch A., Trachsel V., Lutolf M.P. (2015). Brief Report: Single-Cell Analysis Reveals Cell Division-Independent Emergence of Megakaryocytes From Phenotypic Hematopoietic Stem Cells. Stem Cells.

[bib71] Rodriguez-Fraticelli A.E., Wolock S.L., Weinreb C.S., Panero R., Patel S.H., Jankovic M., Sun J., Calogero R.A., Klein A.M., Camargo F.D. (2018). Clonal analysis of lineage fate in native haematopoiesis. Nature.

[bib72] Rodriguez-Meira A., Buck G., Clark S.A., Povinelli B.J., Alcolea V., Louka E., McGowan S., Hamblin A., Sousos N., Barkas N. (2019). Unravelling Intratumoral Heterogeneity through High-Sensitivity Single-Cell Mutational Analysis and Parallel RNA Sequencing. Mol. Cell.

[bib73] Sanada C., Xavier-Ferrucio J., Lu Y.C., Min E., Zhang P.X., Zou S., Kang E., Zhang M., Zerafati G., Gallagher P.G., Krause D.S. (2016). Adult human megakaryocyte-erythroid progenitors are in the CD34+CD38mid fraction. Blood.

[bib74] Sanjuan-Pla A., Macaulay I.C., Jensen C.T., Woll P.S., Luis T.C., Mead A., Moore S., Carella C., Matsuoka S., Bouriez Jones T. (2013). Platelet-biased stem cells reside at the apex of the haematopoietic stem-cell hierarchy. Nature.

[bib75] Senis Y.A., Tomlinson M.G., García A., Dumon S., Heath V.L., Herbert J., Cobbold S.P., Spalton J.C., Ayman S., Antrobus R. (2007). A comprehensive proteomics and genomics analysis reveals novel transmembrane proteins in human platelets and mouse megakaryocytes including G6b-B, a novel immunoreceptor tyrosine-based inhibitory motif protein. Mol. Cell. Proteomics.

[bib76] Shin J.Y., Hu W., Naramura M., Park C.Y. (2014). High c-Kit expression identifies hematopoietic stem cells with impaired self-renewal and megakaryocytic bias. J. Exp. Med..

[bib77] Siripin D., Kheolamai P., U-Pratya Y., Supokawej A., Wattanapanitch M., Klincumhom N., Laowtammathron C., Issaragrisil S. (2015). Transdifferentiation of erythroblasts to megakaryocytes using FLI1 and ERG transcription factors. Thromb. Haemost..

[bib78] Tomer A. (2004). Human marrow megakaryocyte differentiation: multiparameter correlative analysis identifies von Willebrand factor as a sensitive and distinctive marker for early (2N and 4N) megakaryocytes. Blood.

[bib79] Ulveling D., Le Clerc S., Cobat A., Labib T., Noirel J., Laville V., Coulonges C., Carpentier W., Nalpas B., Heim M.H., HEPAVIH ANRS CO13 Cohort Study Group, Swiss Hepatitis C Cohort Study Group, French ANRS HC EP 26 Genoscan Study Group. (2016). A new 3p25 locus is associated with liver fibrosis progression in human immunodeficiency virus/hepatitis C virus-coinfected patients. Hepatology.

[bib80] Velten L., Haas S.F., Raffel S., Blaszkiewicz S., Islam S., Hennig B.P., Hirche C., Lutz C., Buss E.C., Nowak D. (2017). Human haematopoietic stem cell lineage commitment is a continuous process. Nat. Cell Biol..

[bib81] von Kleist L., Stahlschmidt W., Bulut H., Gromova K., Puchkov D., Robertson M.J., MacGregor K.A., Tomilin N., Pechstein A., Chau N. (2011). Role of the clathrin terminal domain in regulating coated pit dynamics revealed by small molecule inhibition. Cell.

[bib82] Wattacheril J., Lavine J.E., Chalasani N.P., Guo X., Kwon S., Schwimmer J., Molleston J.P., Loomba R., Brunt E.M., Chen Y.I. (2017). Genome-Wide Associations Related to Hepatic Histology in Nonalcoholic Fatty Liver Disease in Hispanic Boys. J. Pediatr..

[bib83] Wen Q.J., Yang Q., Goldenson B., Malinge S., Lasho T., Schneider R.K., Breyfogle L.J., Schultz R., Gilles L., Koppikar P. (2015). Targeting megakaryocytic-induced fibrosis in myeloproliferative neoplasms by AURKA inhibition. Nat. Med..

[bib84] Wolf F.A., Angerer P., Theis F.J. (2018). SCANPY: large-scale single-cell gene expression data analysis. Genome Biol..

[bib85] Wolf F.A., Hamey F.K., Plass M., Solana J., Dahlin J.S., Göttgens B., Rajewsky N., Simon L., Theis F.J. (2019). PAGA: graph abstraction reconciles clustering with trajectory inference through a topology preserving map of single cells. Genome Biol..

[bib86] Wright F.A., Strug L.J., Doshi V.K., Commander C.W., Blackman S.M., Sun L., Berthiaume Y., Cutler D., Cojocaru A., Collaco J.M. (2011). Genome-wide association and linkage identify modifier loci of lung disease severity in cystic fibrosis at 11p13 and 20q13.2. Nat. Genet..

[bib87] Yamamoto R., Morita Y., Ooehara J., Hamanaka S., Onodera M., Rudolph K.L., Ema H., Nakauchi H. (2013). Clonal analysis unveils self-renewing lineage-restricted progenitors generated directly from hematopoietic stem cells. Cell.

