# Supplementary material for: Single-Cell Analyses Reveal Megakaryocyte-Biased Hematopoiesis in Myelofibrosis and Identify Mutant Clone-Specific Targets
Source: Mol Cell. 2020 May 7;78(3):477–492.e8. doi: 10.1016/j.molcel.2020.04.008 (PMC7217381; doi:10.1016/j.molcel.2020.04.008)
Supplement: Document S1. Figures S1–S7 and Tables S1, S5, and S7 [file mmc1.pdf]

Molecular Cell, Volume *78*

Supplemental Information

Single-Cell Analyses Reveal Megakaryocyte-Biased Hematopoiesis in Myelofibrosis and Identify Mutant Clone-Specific Targets

Bethan Psaila, Guanlin Wang, Alba Rodriguez-Meira, Rong Li, Elisabeth F. Heuston, Lauren Murphy, Daniel Yee, Ian S. Hitchcock, Nikolaos Sousos, Jennifer O'Sullivan, Stacie Anderson, Yotis A. Senis, Olga K. Weinberg, Monica L. Calicchio, NIH Intramural Sequencing Center, Deena Iskander, Daniel Royston, Dragana Milojkovic, Irene Roberts, David M. Bodine, Supat Thongjuea, and Adam J. Mead

SUPPLEMENTAL ITEMS (Psaila *et al)*

Supplemental Figure Legends

Suppl. Figure 1, Relating to Figures 1-7 and methods. Isolation and characterization of CD41+ and CD41- HSPCs, integration of scRNAseq data from individual donors and cell cluster identification. S1A. $Lin^{-}CD34^{+}CD38^{-}CD45RA^{-}CD41^{+}$ (CD41+ HSC/MPP) and $Lin^{-}CD34^{+}CD38^{+}CD45RA^{-}CD41^{+}$ (CD41+ CMP/MEP) compartments contain cells with typical blast cell morphology and not mature megakaryocytes. Cells were isolated by FACS and stained with May Grunewald Giemsa, representative cells shown (200X magnification). **S1B.** Single-cell colony output from control and myelofibrosis CD41-HSC, CD41-MPP and $CD41^{+}$HSC/MPP cells FACS-isolated into individual wells of 96-well plates containing methylcellulose. Total colonies counted = 250 sorted from healthy control (n=5) and myelofibrosis (n=6) donors. Abbreviations: CFU – colony-forming unit; GM – granulocyte-monocyte; E – erythroid; GEMM – granulocyte-erythroid-monocyte-megakaryocyte. * - P<0.05 for CFU-GM output of control *vs*. myelofibrosis CD41- MPP; all other comparisons non-significant. **S1C:** All 122,154 cells were aggregated from the 21 individual donors, with good integration of samples and batches after individual donor effect was regressed out. Left plot - cells are colored by donor ID; right plot – cells colored by batch. **S1D:** A cluster of cells expressing high levels of plasmacytoid dendritic cell markers *GZMB*, *EPHA2* and *PPM1J* was identified and removed from downstream analyses, as these are mature lymphoid cells and not hematopoietic stem/progenitor cells. **S1E:** Marker genes for the 8 identified clusters in the UMAP aggregate of all cells. **S1F:** Myelofibrosis cells were down-sampled to generate an aggregate that contained equal numbers of myelofibrosis and control cells (37,941 cells of each). Left plot – distribution of cells by disease (myelofibrosis – red; controls – blue); middle – superimposition of lineage signature genes (Suppl. Table 5); right – lineage progenitor clusters (Mk – megakaryocyte; Lymph – lymphoid; Ery – erythroid; Mye – myeloid), relating to Fig. 2D.

Suppl. Fig. 2, relating to Figures 2 and 3 and Suppl. Table 5. Expression of individual genes comprising lineage signature gene sets. Cells on Uniform Manifold Approximation and Projection (UMAP) plots are colored according to expression levels of each gene used in the respective lineage progenitor 'gene signature' set. Abbreviations: HSPC - hematopoietic stem/progenitor cells; Ery – eythroid; Lym – lymphoid; Mega – megakaryocyte; Mye – myeloid.

Suppl. Fig. 3, Relating to Figures 2 and 3. Force-directed trajectory graphs (FDG) for individual donors and comparison of FDG and diffusion map trajectory analyses. **S3A:** Patients with

JAK2V617F-mutated myelofibrosis (n=12); **S3B:** Patients with *CALR*-mutated myelofibrosis (n=3); **S3C:** Healthy donor controls (n=6). Diagnosis (primary or secondary myelofibrosis) and treatment at time of analysis are shown (PPV MF – post polycythemia vera myelofibrosis; PET MF – post essential thrombocythemia myelofibrosis; PMF – primary myelofibrosis; Hydroxy – hydroxycarbamide; No Tx – no treatment; Rux – ruxolitinib. **S3D:** Differentiation trajectory plots generated using Scanpy confirms presence of substantially augmented megakaryocyte differentiation trajectory as seen in FDG (Fig. 3) generated using in-house pipeline. Left plots – UMAP; middle – FDG, Right – diffusion maps. Arrow indicates megakaryocyte trajectory in each plot.

Suppl. Fig. 4, Relating to Fig. 5. Identification and comparison of myelofibrosis and control megakaryocyte progenitors (MkP). S4A: UMAP plots of control (top row) and myelofibrosis (MF, bottom row) HSPCs. Far left – expression of lineage gene scores; Left – identified clusters; Right – cells with an AUCell score for Mk genes >0.4; Far right –overlap, i.e. all cells from clusters with a Mk gene expression signature (clusters 8, 13, 14, 15, 16 for MF and 13 and 14 for controls) that had an AUCell score > 0.4. **S4B:** Heatmap of all differentially expressed genes (n=3,496) demonstrating that the majority of control MkP have a similar transcriptional profile to one sub-cluster of myelofibrosis MkP, while a larger unique population of MF MkP was detected with overall upregulation of gene expression.

Suppl. Fig. 5, relating to Fig. 5. Marker genes for myelofibrosis megakaryocyte progenitor sub-clusters and cell cycle genes. S5A: Heatmap showing 6 differentially expressed genes for each of the myelofibrosis MkP sub-clusters. **S5B:** Expression of G2M and S-phase genes on the myelofibrosis HSPC (left) and MkP (right) UMAPs. **S5C:** Expression of *MKI67* in the UMAPS for myelofibrosis (left) and control (right) HSPCs.

Suppl. Fig. 6, Relating to Figure 6. **Trajectory analysis of the integrated dataset of cells analysed by 10x Genomics and TARGET-seq (simultaneous mutation detection and scRNAseq) and *G6B* expression within distinct molecular sub-clones. S6A**: Top left: Force directed graph (FDG) showing integration of cells analysed by 10x Genomics and TARGET-seq; Top right: FDG with lineage gene signature scores overlaid; lower left: unsupervised cell clustering (Louvain); lower right: cells analysed by TARGET-seq identified by genotypte (wild-type and mutant); chart shows percentage of wild-type and mutant cells in the louvain clusters corresponding to the lineage progenitors (myeloid – cluster 7; megakaryocyte (MK) – cluster 4; erythroid – cluster 3). P-values shown for Fisher's exact test for relevant pair-wise comparison (* - $P<0.05$; *** - $P<0.001$). **S6B:** Expression of *G6B* is detected

in all genetic subclones in two myelofibrosis patients with 3 or more genetic subclones. x-axis – number of cells in which *G6B* expression was detected out of all cells of each genetic subclone identified. All pair-wise comparisons non-significant (Fisher's Exact Test).

Suppl. Fig. 7, Relating to Figure 7. Expression and targeting of *G6B* in *JAK2V617F*-mutated MPN cell lines and other leukemic cell lines. **S7A:** Expression and frequency of selected cell surface markers in MARIMO (mut*CALR* acute myeloid leukemia), HL60 (acute myeloid leukemia), JURKAT (T-cell leukemia), K562 (chronic myeloid leukemia), or human embryonic kidney HEK cells measured by CyTOF. S7B: Live cell imaging (left) and flow cytometry demonstrating internalization of CD34 x G6B bispecific in HEL cells (which are CD34–, G6B+). N=3.

Supplemental Tables 1, 5 and 7 (Suppl. Tables 2, 3, 4 and 6 as excel files)

Suppl. Table 1, Relating to Figures 1-7: Clinical details of all myelofibrosis patients and healthy donor controls studied.

Abbreviations: PMF – primary myelofibrosis; PET MF – post essential thrombocythemia myelofibrosis; PPV MF – post polycythaemia myelofibrosis; Con – healthy donor controls; Hydroxy – hydroxycarbamide; EPO – recombinant human erythropoietin. DIPPS – dynamic international prognostic scoring system (Passamonti et al., 2010).

Study ID	Age	Sex	Disease status	Mutations	Treatment	WHO fibrosis grade in BM	DIPPS risk group
001_001	70	M	PMF	*JAK2V617F, CBL, SRSF2*	None	3	Int-1
001_002	56	M	PET MF	*CALR type 2, U2AF1*	Ruxolitinib	3	Int-2
001_018	73	M	PPV MF	*JAK2V617F, ASXL1, ETV6*	None	3	Int-2
001_020	61	M	PET MF	*CALR type 1*	Ruxolitinib	3	Low
001_023	86	M	PMF	*JAK2V617F, SF3B1*	Ruxolitinib	3	Int-2
001_038	60	M	PPV MF	*JAK2V617F*	Hydroxy	3	int-1
001_040	73	F	PPV MF	*JAK2V617F*	Ruxolitinib	3	Int-2
001_042	57	F	PET MF	*MPL W515K*	None	2-3	Int-1

001_047	81	F	PMF	*CALR type 1*	Ruxolitinib	3	Int-1
001_054	76	F	PET MF	*JAK2V617F, ETV6*	None (prior anagrelide)	3	Int-2
001_057	67	F	PPV MF	*JAK2V617F*	Ruxolitinib	2	Int-1
001_076	70	F	PET MF	*CALR type 1*	Luspatercept	2	Int-1
001_098	58	F	PMF	*CALR type 2, ASXL1*	None	2-3	Int-1
001_102	72	F	PMF	*CALR type 1*	Pacritinib	2	Int-1
001_110	69	M	PPV MF	*JAK2V617F, ASXL1, EZH2*	None	3	Int-2
001_114	68	M	PPV MF	*JAK2V617F, ASXL1*	Hydroxy	3	Int-1
001_124	70	M	PMF	*JAK2V617F, ASXL1*	Ruxolitinib	3	Int-2
001_127	61	M	PMF	*JAK2V617F, TET2, SF3B1*	None	3	Int-2
001_128	54	M	PMF	*CALR Type 1-like*	None	3	Int-1
001_131	49	F	PPV MF	*JAK2V617F*	None	2-3	Int-1
001_132	75	M	PPV MF	*JAK2V617F*	Ruxolitinib	3	Int-2
006_002	67	F	PMF	*JAK2V617F*	Momelotinib	3	Int-1
010_022	65	F	PPV MF	*JAK2V617F*	Hydroxy	2	Int-1
010_054	77	M	PPV MF	*JAK2V617F*	Ruxolitinib	3	Int-2
010_047	56	F	PMF	*JAK2V617F*	Ruxolitinib	3	Int-2
010_005	62	M	PMF	*JAK2V617F*	Danazol	3	Int-2
010_027	72	M	PET MF	*JAK2V617F*	Ruxolitinib and EPO	2	Int-2
010_028	74	M	PPV MF	*JAK2V617F*	Ruxolitinib	2	Int-1
010_003	79	M	PPV MF	*JAK2V617F*	Ruxolitinib	3	Int-2
010_055	54	F	PMF	*JAK2V617F*	Ruxolininib	2	Int-1
010_019	78	M	PET MF	*JAK2V617F*	Ruxolitinib and EPO	3	Int-2
010_056	60	M	PMF	*JAK2V617F*	None	3	Int-1
009_001	67		Control				
009_002	42		Control				
009_003	35		Control				
009_005	60		Control				

009_007	56		Control				
009_008	55		Control				
014_001	44		Control				
014_002	26		Control				
014_003	32		Control				
010_9001	54		Control				
010_9002	58		Control				
010_9003	42		Control				
010_9004	44		Control				
010_9005	69		Control				
011_001	40		Control				
012_9001	44		Control				
012_9002	53		Control				
012_9003	41		Control				
012_9004	33		Control				
012_9005	28		Control				
012_9006	51		Control				
007_002	61		Control				
007_003	78		Control				
007_005	73		Control				

Suppl. Table 5, Relating to Figures 2, 3 and 5. Genes included in HSPC/lineage progenitor gene signatures, fibrosis signature gene sets and G2M and S phase gene sets.

**Mye**	**Ery**	**Lymph**	**Mega**	**HSPC**	**Fibrosis Signature Gene Score**	**G2M**	**S phase**
ELANE	*EPOR*	*VPREB1*	*MPIG6B*	*CRHBP*	*TGFB1*	*HMGB2*	*MCM5*
AZU1	*KLF1*	*JCHAIN*	*PF4*	*EMCN*	*IL12A*	*CDK1*	*PCNA*
PRTN3	*TFR2*	*CD22*	*GP9*	*HLF*	*IL15*	*NUSAP1*	*TYMS*
CFD	*CSF2RB*	*IGHD*	*VWF*	*AVP*	*IL1B*	*UBE2C*	*FEN1*
MPO	*APOE*	*LTB*	*SELP*		*ACTR5*	*BIRC5*	*MCM2*
CSF1R	*APOC1*				*RAB37*	*TPX2*	*MCM4*
CST7	*CNRIP1*				*C20orf194*	*TOP2A*	*RRM1*
CTSG					*TOLLIP*	*NDC80*	*UNG*
					GOSR2	*CKS2*	*GINS2*

					TIMP1	*NUF2*	*MCM6*
					APIP	*CKS1B*	*CDCA7*
					RAB7B	*MKI67*	*DTL*
					PF4	*TMPO*	*PRIM1*
					VEGFA	*CENPF*	*UHRF1*
						TACC3	*MLF1IP*
						FAM64A	*HELLS*
						SMC4	*RFC2*
						CCNB2	*RPA2*
						CKAP2L	*NASP*
						CKAP2	*RAD51AP1*
						AURKB	*GMNN*
						BUB1	*WDR76*
						KIF11	*SLBP*
						ANP32E	*CCNE2*
						TUBB4B	*UBR7*
						GTSE1	*POLD3*
						KIF20B	*MSH2*
						HJURP	*ATAD2*
						CDCA3	*RAD51*
						HN1	*RRM2*
						CDC20	*CDC45*
						TTK	*CDC6*
						CDC25C	*EXO1*
						KIF2C	*TIPIN*
						RANGAP1	*DSCC1*
						NCAPD2	*BLM*
						DLGAP5	*CASP8AP2*
						CDCA2	*USP1*
						CDCA8	*CLSPN*
						ECT2	*POLA1*
						KIF23	*CHAF1B*
						HMMR	*BRIP1*
						AURKA	*E2F8*
						PSRC1	
						ANLN	
						LBR	
						CKAP5	
						CENPE	
						CTCF	
						NEK2	

						G2E3	
						GAS2L3	
						CBX5	
						CENPA	

Suppl. Table 7, Relating to Figure 7. Antibodies used for cell surface antigens in CyTOF panel.

**Marker**	**Clone**	**Supplier**	**Catalogue number**	**Titration (uL/100ul)**	**Isotope**
CD41	HIP8	Fluidigm	3089004B	1.0	89Y
CD19	SJ25-C1	Invitrogen	MHCD1906	0.2	111Cd
CD9	HI9a	Biolegend	312102-B227707	1.0	142 Nd
CD45RA	HI100	Fluidigm	3143006B	1.0	143 Nd
CD42b	HIP1	Fluidigm	3144020B	1.0	144 Nd
CD4	RPA-T4	Fluidigm	300502	0.5	145 Nd
CD34	581	Fluidigm	3148001B	1.0	148 Nd
CD56	NCAM16.3	Fluidigm	3149021B	1.0	149 Sm
CD123 (IL-3R)	6H6	Fluidigm	3151001B	1.0	151 Eu
CD3	UCHT1	Fluidigm	3154003B	0.5	154 Sm
CD36	5-271	Fluidigm	3155012B	1.0	155 Gd
CD14	M5E2	Biolegend	301802	0.3	160 Gd
CD90	5.00E+10	Fluidigm	3161009B	1.0	161 Dy
CD49f	G0H3	Fluidigm	3164006B	1.0	164 Dy
CD44	BJ18	Fluidigm	3166001B	1.0	166 Er
RXFP1	933344	R&D Systems	MAB8898	1.0	170 Er
CD38	HIT2	Fluidigm	3172007B	1.0	172 Yb
CLEC2	AYP1	Biolegend	372002	1.0	174 Yb
CD71	OKT-9	Fluidigm	3175011B	1.0	175 Lu
G6B	clone 17-4	Collaborator	NA	2.0	176 Yb

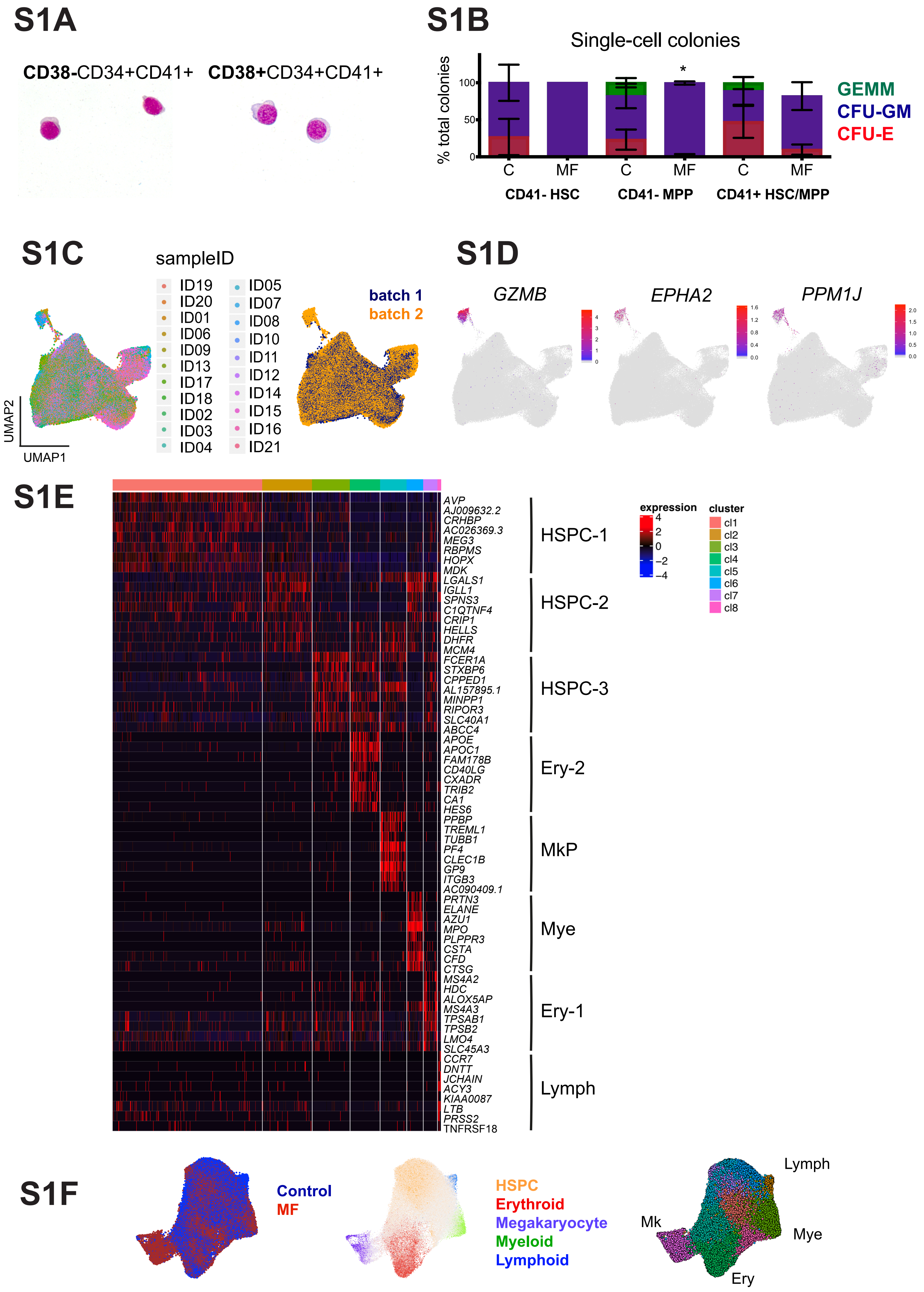
S1A
CD38-CD34+CD41+
CD38+CD34+CD41+
S1B
Single-cell colonies
% total colonies
100
50
0
*
C
MF
C
MF
C
MF
CD41- HSC
CD41- MPP
CD41+ HSC/MPP
GEMM
CFU-GM
CFU-E
S1C
sampleID
ID19
ID20
ID01
ID06
ID09
ID13
ID17
ID18
ID02
ID03
ID04
ID05
ID07
ID08
ID10
ID11
ID12
ID14
ID15
ID16
ID21
UMAP2
UMAP1
batch 1
batch 2
S1D
GZMB
EPHA2
PPM1J
S1E
AVP
AJ009632.2
CRHBP
AC026369.3
MEG3
RBPMS
HOPX
MDK
LGALS1
IGLL1
SPNS3
C1QTNF4
CRIP1
HELLS
DHFR
MCM4
FCER1A
STXBP6
CPPED1
AL157895.1
MINPP1
RIPOR3
SLC40A1
ABCC4
APOE
APOC1
FAM178B
CD40LG
CXADR
TRIB2
CA1
HES6
PPBP
TREML1
TUBB1
PF4
CLEC1B
GP9
ITGB3
AC090409.1
PRTN3
ELANE
AZU1
MPO
PLPPR3
CSTA
CFD
CTSG
MS4A2
HDC
ALOX5AP
MS4A3
TPSAB1
TPSB2
LMO4
SLC45A3
CCR7
DNTT
JCHAIN
ACY3
KIAA0087
LTB
PRSS2
TNFRSF18
HSPC-1
HSPC-2
HSPC-3
Ery-2
MkP
Mye
Ery-1
Lymph
expression
4
2
0
−2
−4
cluster
cl1
cl2
cl3
cl4
cl5
cl6
cl7
cl8
S1F
Control
MF
HSPC
Erythroid
Megakaryocyte
Myeloid
Lymphoid
Lymph
Mk
Mye
Ery

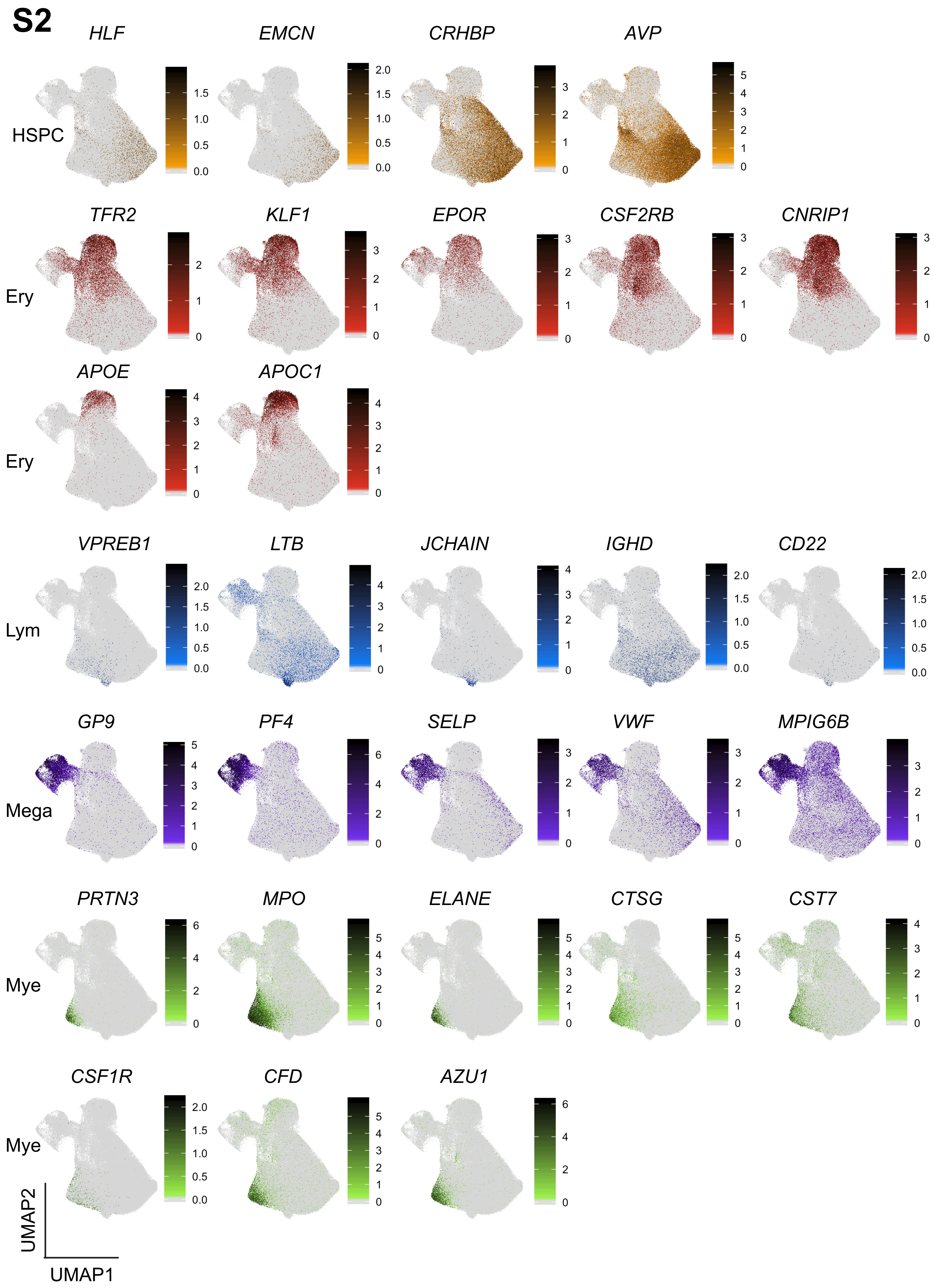

S2
HLF
EMCN
CRHBP
AVP
HSPC
TFR2
KLF1
EPOR
CSF2RB
CNRIP1
Ery
APOE
APOC1
Ery
VPREB1
LTB
JCHAIN
IGHD
CD22
Lym
GP9
PF4
SELP
VWF
MPIG6B
Mega
PRTN3
MPO
ELANE
CTSG
CST7
Mye
CSF1R
CFD
AZU1
Mye
UMAP2
UMAP1

S3A *JAK2V617F*+ (N = 12)

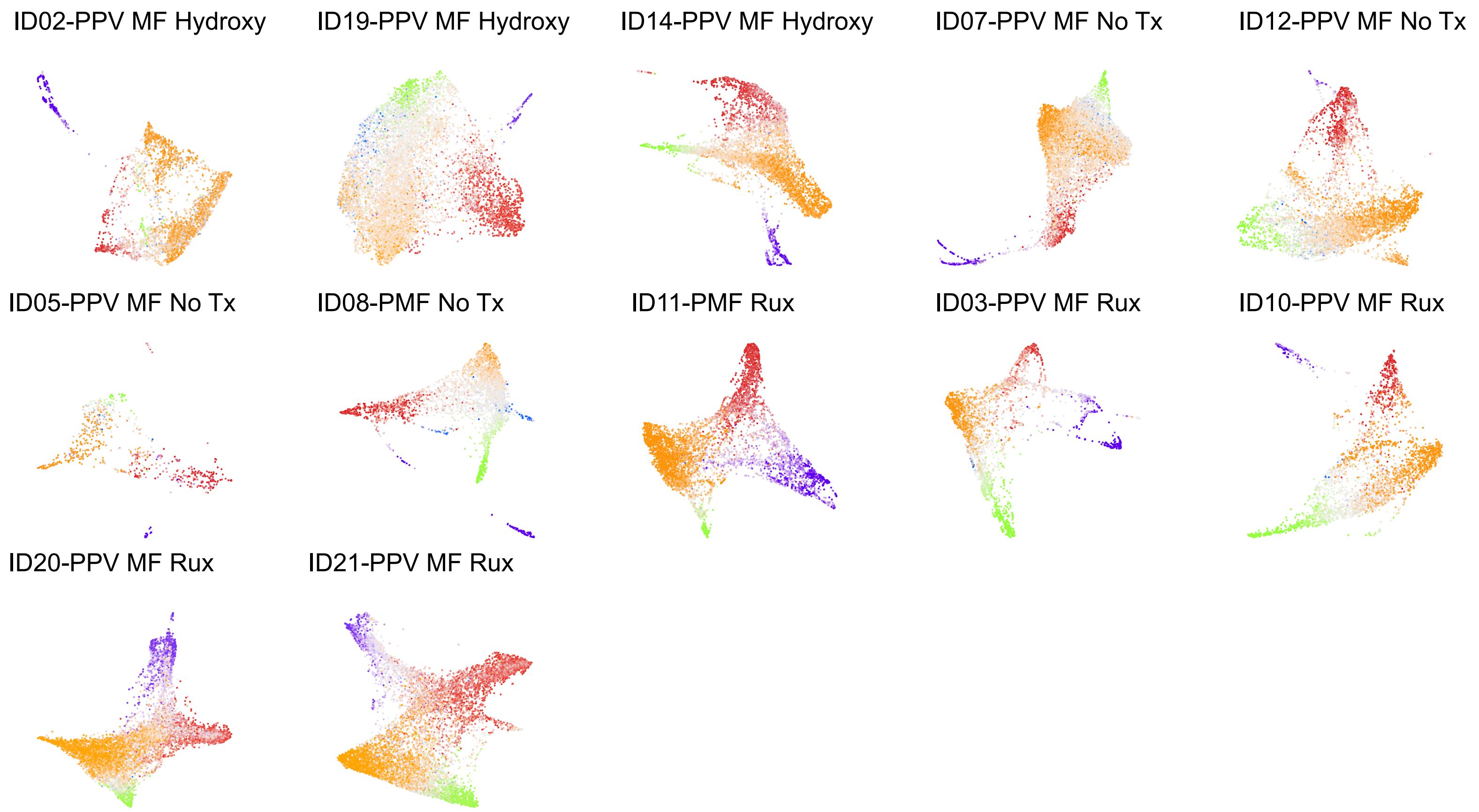

S3B *CALRmut* (N = 3)

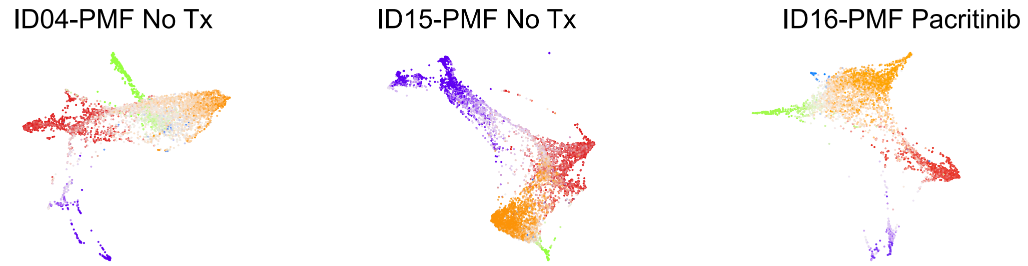

S3C healthy donors (N = 6)

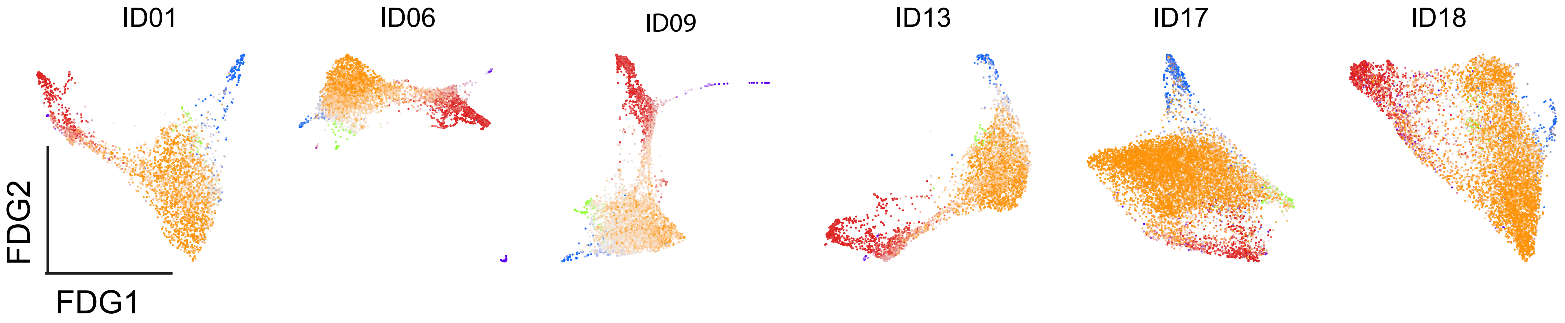

S3D

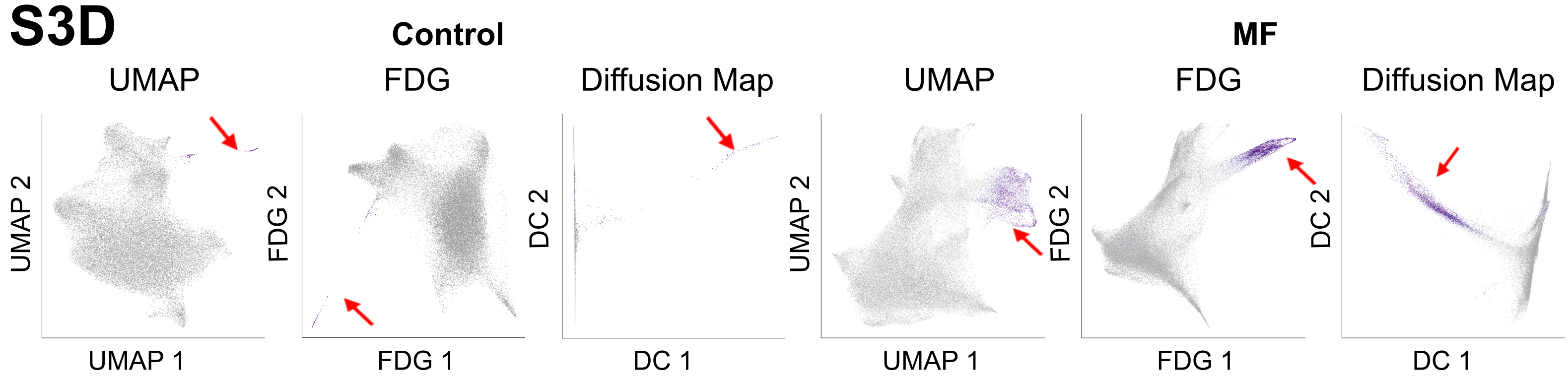

S4A

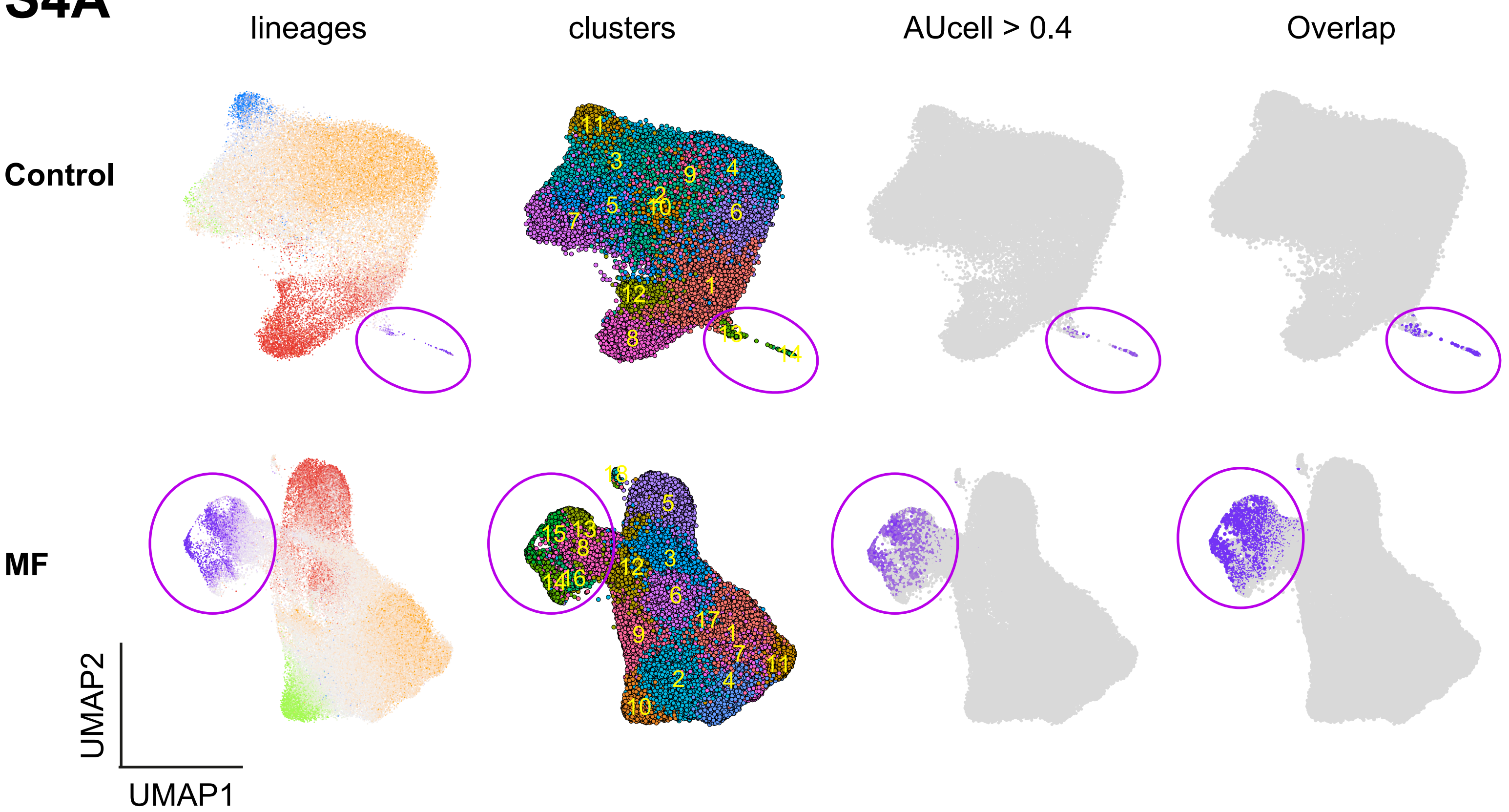
lineages
clusters
AUcell > 0.4
Overlap
Control
MF
UMAP2
UMAP1

S4B

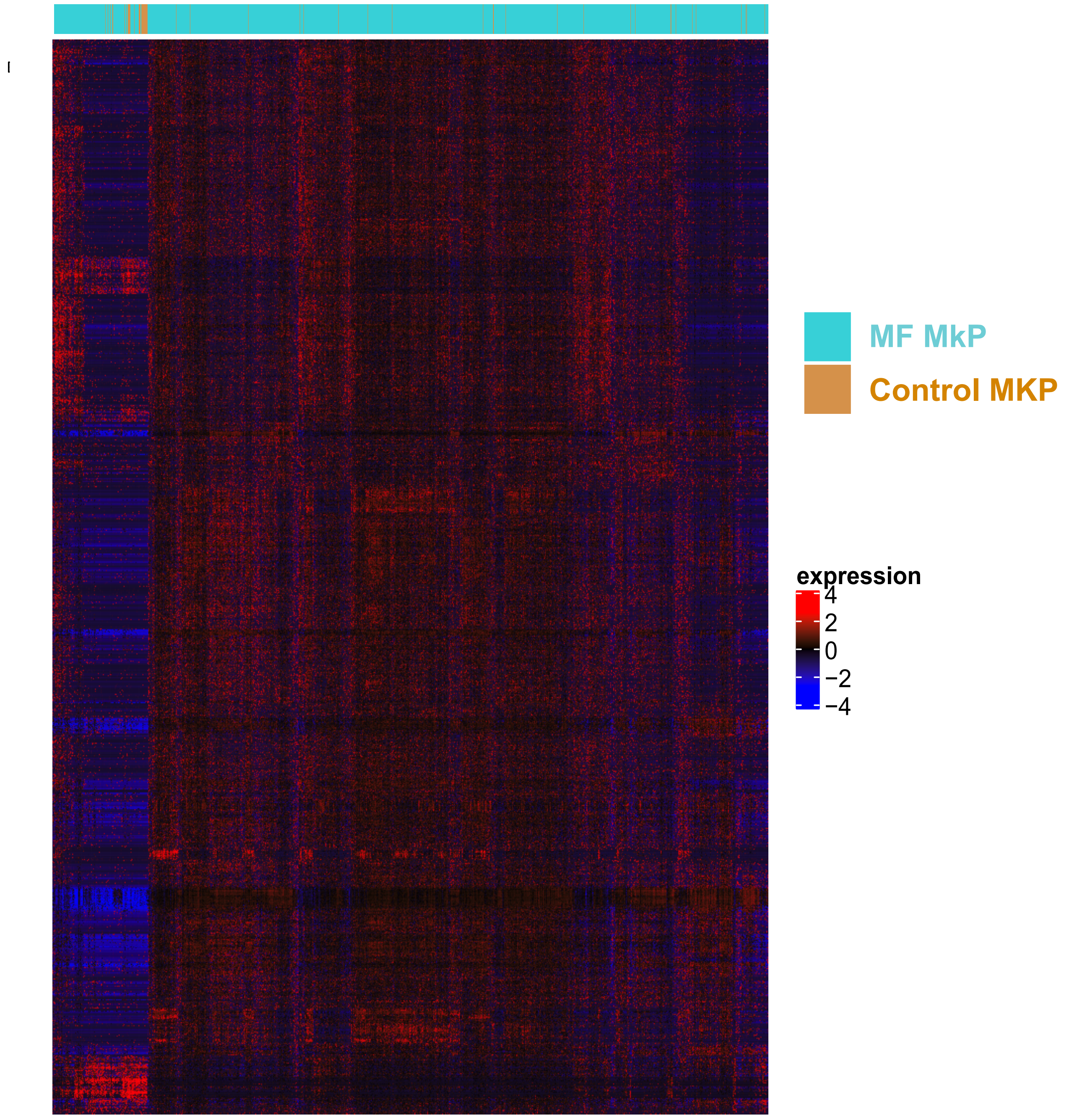
MF MkP
Control MKP
expression
4
2
0
−2
−4

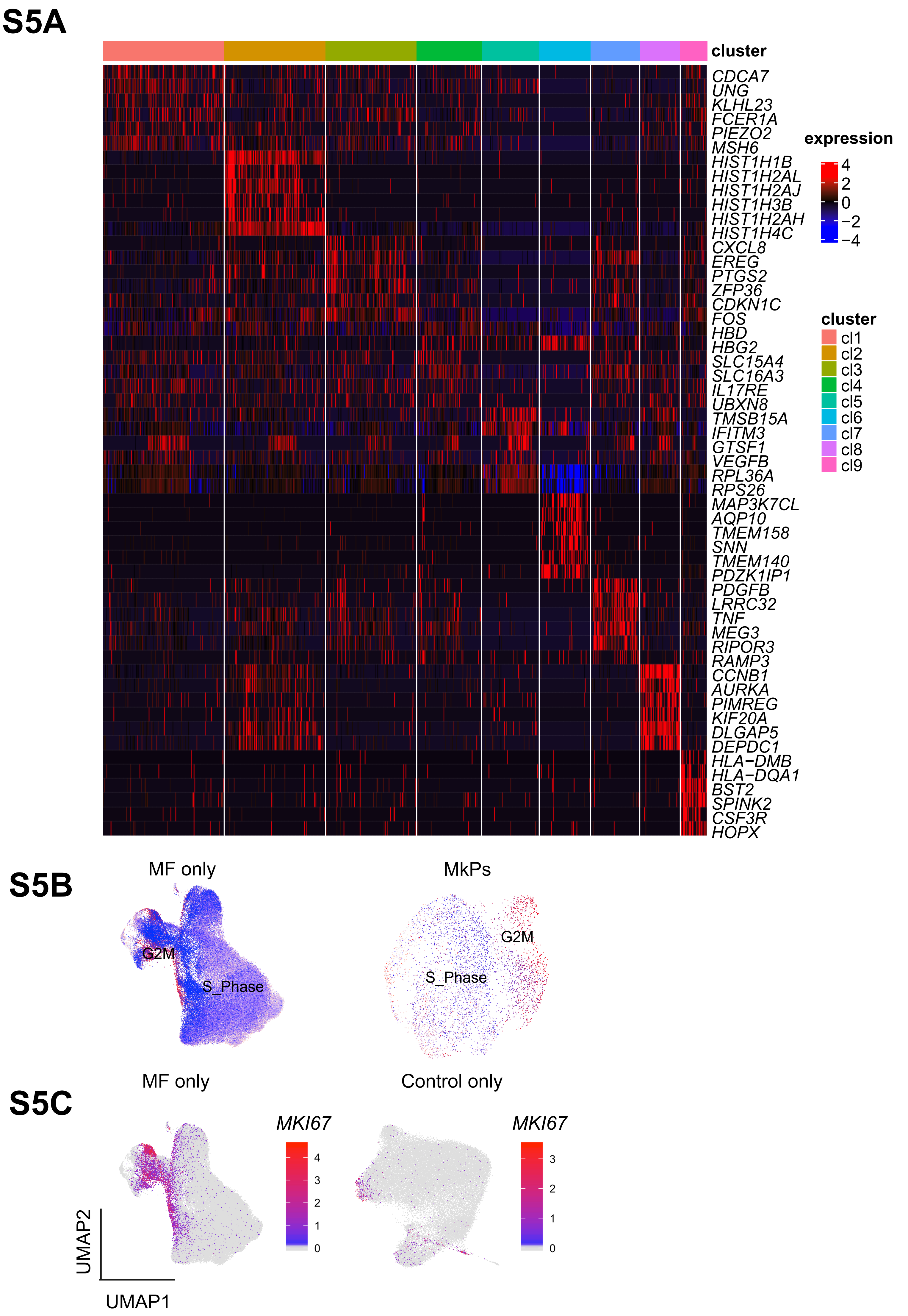
S5A
cluster
CDCA7
UNG
KLHL23
FCER1A
PIEZO2
MSH6
HIST1H1B
HIST1H2AL
HIST1H2AJ
HIST1H3B
HIST1H2AH
HIST1H4C
CXCL8
EREG
PTGS2
ZFP36
CDKN1C
FOS
HBD
HBG2
SLC15A4
SLC16A3
IL17RE
UBXN8
TMSB15A
IFITM3
GTSF1
VEGFB
RPL36A
RPS26
MAP3K7CL
AQP10
TMEM158
SNN
TMEM140
PDZK1IP1
PDGFB
LRRC32
TNF
MEG3
RIPOR3
RAMP3
CCNB1
AURKA
PIMREG
KIF20A
DLGAP5
DEPDC1
HLA−DMB
HLA−DQA1
BST2
SPINK2
CSF3R
HOPX
expression
4
2
0
−2
−4
cluster
cl1
cl2
cl3
cl4
cl5
cl6
cl7
cl8
cl9
S5B
MF only
G2M
S_Phase
MkPs
G2M
S_Phase
S5C
MF only
MKI67
4
3
2
1
0
Control only
MKI67
3
2
1
0
UMAP2
UMAP1

S6A

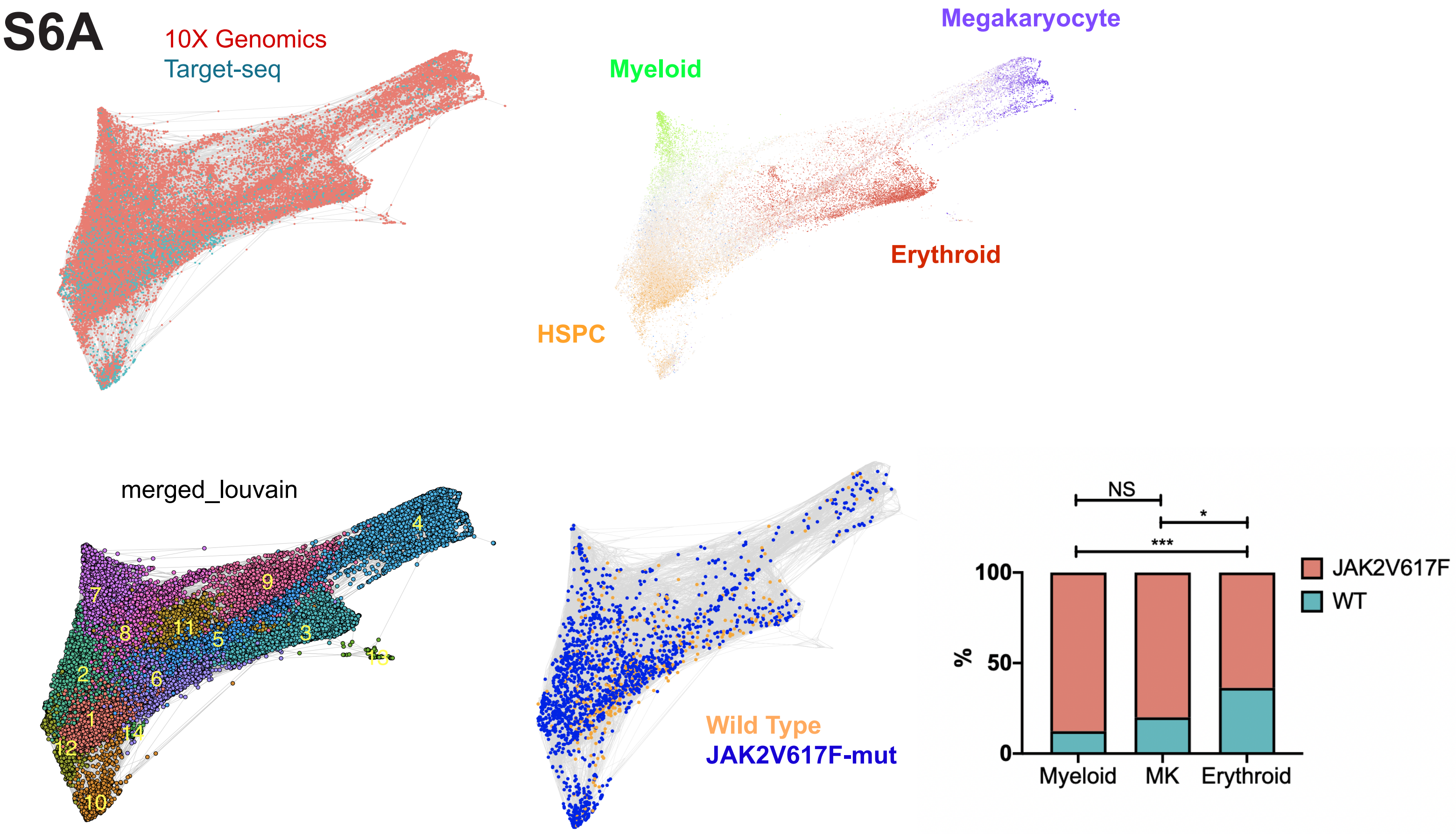
10X Genomics
Target-seq
Myeloid
Megakaryocyte
Erythroid
HSPC
merged_louvain
Wild Type
JAK2V617F-mut
NS
*
***
100
50
0
%
Myeloid
MK
Erythroid
JAK2V617F
WT

S6B

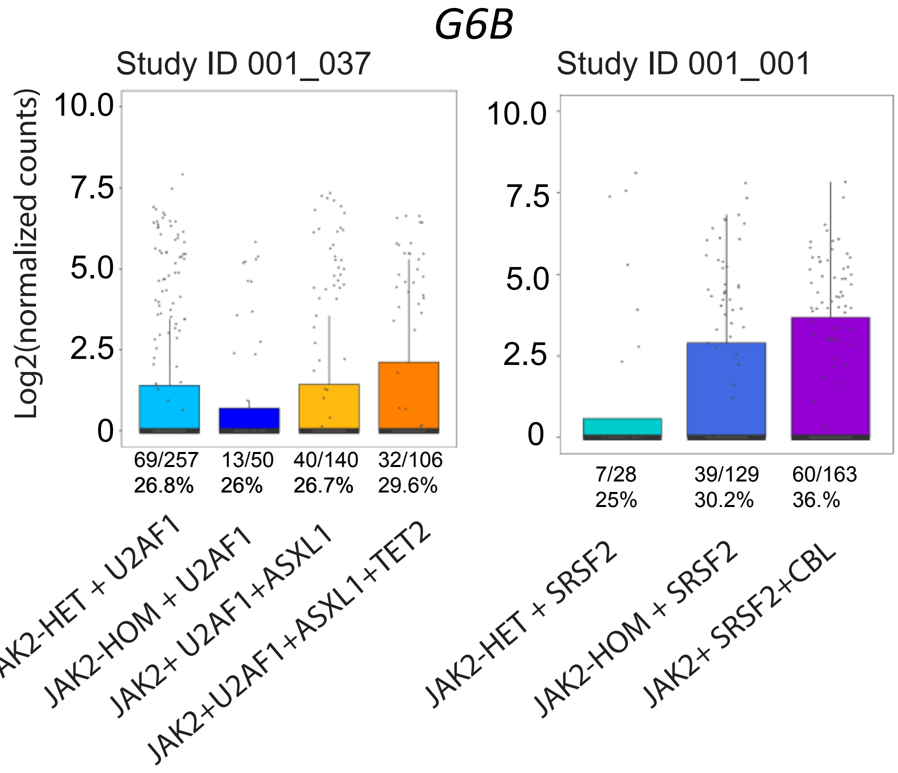
G6B
Study ID 001_037
Study ID 001_001
Log2(normalized counts)
10.0
7.5
5.0
2.5
0
69/257
26.8%
13/50
26%
40/140
26.7%
32/106
29.6%
7/28
25%
39/129
30.2%
60/163
36.%
JAK2-HET + U2AF1
JAK2-HOM + U2AF1
JAK2+ U2AF1+ASXL1
JAK2+U2AF1+ASXL1+TET2
JAK2-HET + SRSF2
JAK2-HOM + SRSF2
JAK2+ SRSF2+CBL

S7A

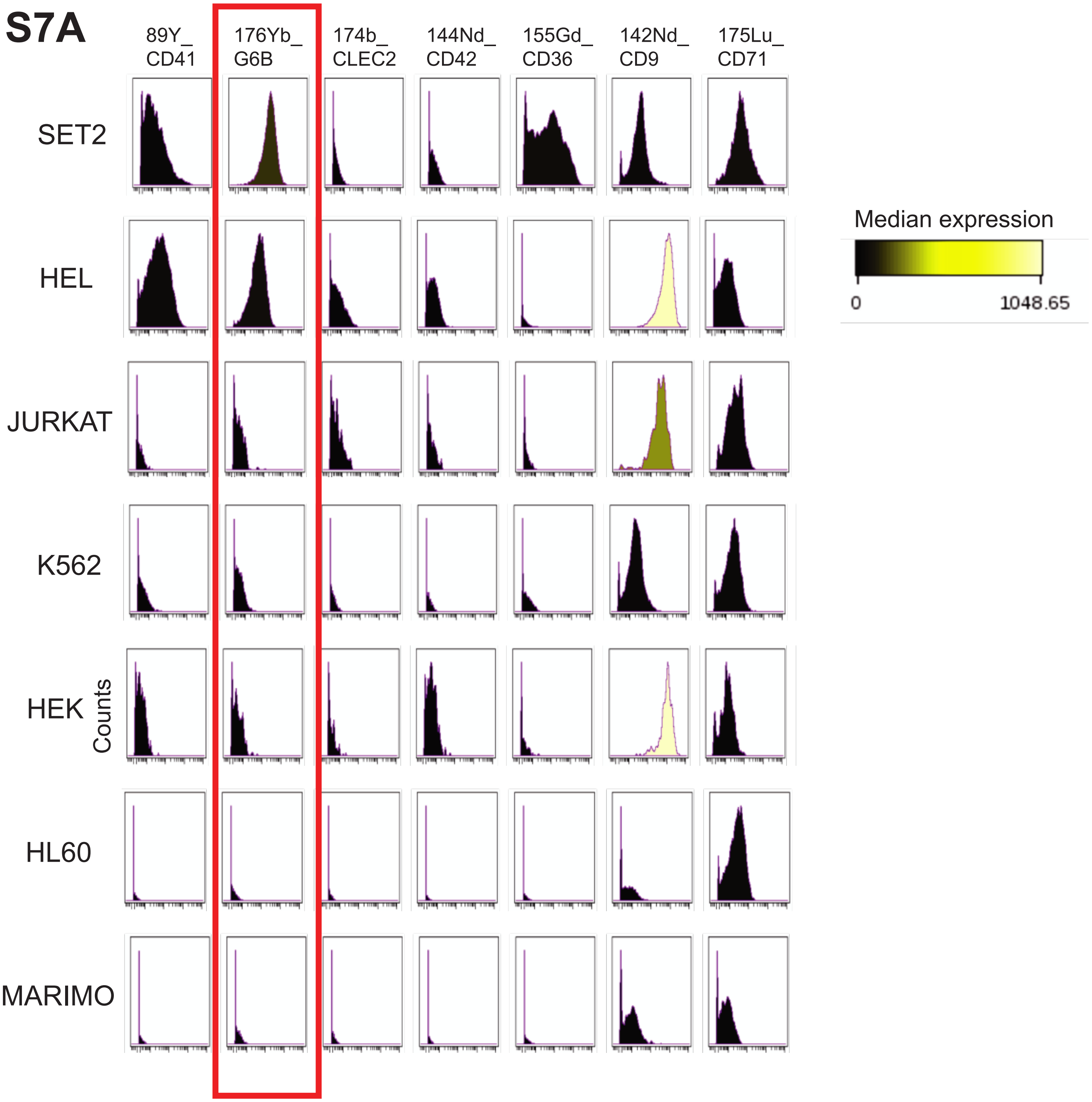

89Y_ CD41
176Yb_ G6B
174b_ CLEC2
144Nd_ CD42
155Gd_ CD36
142Nd_ CD9
175Lu_ CD71
SET2
HEL
JURKAT
K562
HEK
HL60
MARIMO
Counts
Median expression
0
1048.65

S7B

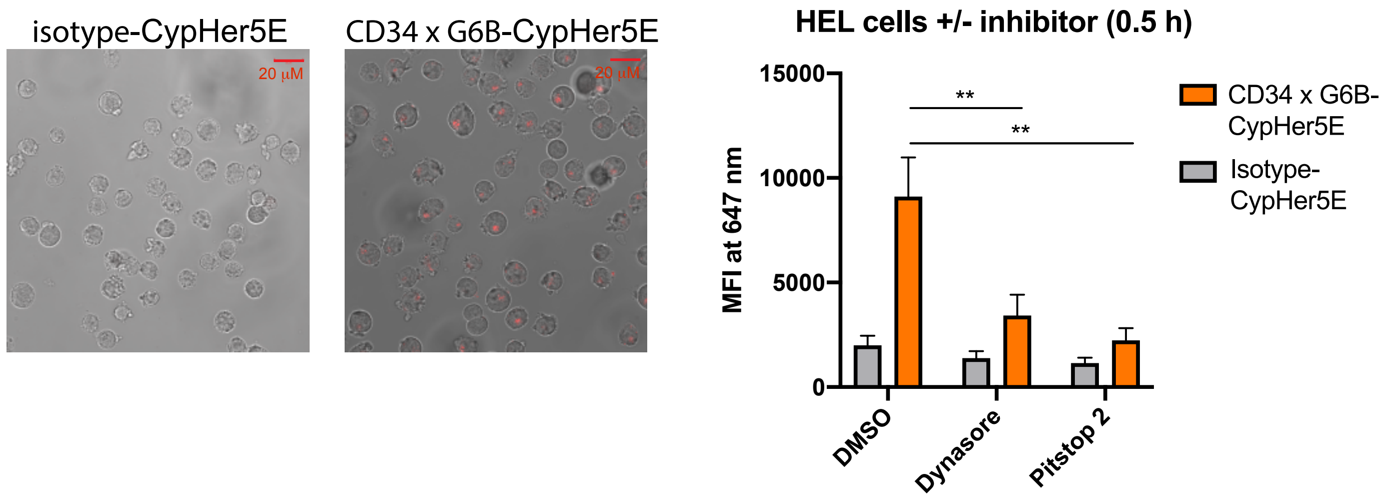

isotype-CypHer5E
CD34 x G6B-CypHer5E
20 µM
20 µM
HEL cells +/- inhibitor (0.5 h)
15000
10000
5000
0
MFI at 647 nm
**
**
DMSO
Dynasore
Pitstop 2
CD34 x G6B-CypHer5E
Isotype-CypHer5E
